# Strand Invasion Based Amplification (SIBA®): A Novel Isothermal DNA Amplification Technology Demonstrating High Specificity and Sensitivity for a Single Molecule of Target Analyte

**DOI:** 10.1371/journal.pone.0112656

**Published:** 2014-11-24

**Authors:** Mark J. Hoser, Hannu K. Mansukoski, Scott W. Morrical, Kevin E. Eboigbodin

**Affiliations:** 1 Molecular Biology, GeneForm Technologies, Broadstairs, United Kingdom; 2 Medical communication, Pfizer, Sandwhich, United Kingdom; 3 Department of Biochemistry, University of Vermont, Burlington, United States of America; 4 Research and Development, Orion Diagnostica, Espoo, Finland; Faculty of Biochemistry Biophysics and Biotechnology, Jagiellonian University, Poland

## Abstract

Isothermal nucleic acid amplification technologies offer significant advantages over polymerase chain reaction (PCR) in that they do not require thermal cycling or sophisticated laboratory equipment. However, non-target-dependent amplification has limited the sensitivity of isothermal technologies and complex probes are usually required to distinguish between non-specific and target-dependent amplification. Here, we report a novel isothermal nucleic acid amplification technology, Strand Invasion Based Amplification (SIBA). SIBA technology is resistant to non-specific amplification, is able to detect a single molecule of target analyte, and does not require target-specific probes. The technology relies on the recombinase-dependent insertion of an invasion oligonucleotide (IO) into the double-stranded target nucleic acid. The duplex regions peripheral to the IO insertion site dissociate, thereby enabling target-specific primers to bind. A polymerase then extends the primers onto the target nucleic acid leading to exponential amplification of the target. The primers are not substrates for the recombinase and are, therefore unable to extend the target template in the absence of the IO. The inclusion of 2′-O-methyl RNA to the IO ensures that it is not extendible and that it does not take part in the extension of the target template. These characteristics ensure that the technology is resistant to non-specific amplification since primer dimers or mis-priming are unable to exponentially amplify. Consequently, SIBA is highly specific and able to distinguish closely-related species with single molecule sensitivity in the absence of complex probes or sophisticated laboratory equipment. Here, we describe this technology in detail and demonstrate its use for the detection of *Salmonella.*

## Introduction

The polymerase chain reaction (PCR) revolutionized the field of molecular diagnostics and biological research by allowing specific genes or nucleotide sequences present in sample material to be amplified to detectable levels within approximately 2 hours. More recently, numerous isothermal amplification technologies, which obviate the use of the sophisticated laboratory hardware required for thermal cycling, have been developed [1], [2], [3]. A disadvantage of isothermal technologies is that, in common with PCR, they tend to produce non-specific amplification products. Non-specific amplification results either from binding of the primers to non-target nucleic acids or from direct copying of one primer onto another, a phenomenon known as primer dimers [4]. These events result in non-specific exponential amplification and non-productive consumption of assay components, leading to reduced yield, sensitivity, and/or specificity. In contrast to PCR, the rate of artifactual amplification in isothermal technologies is not aligned with that of the target region throughout the thermal cycle. This can result in non-target-specific products being more rapidly amplified than the target and subsequently overwhelming the reaction, with a further reduction in sensitivity [5], [6].

To retain specificity, several isothermal amplification methods, including HDA [7], RPA [8], NASBA [9], QPA [10], RCA [11], SDA [6], and NEAR/EXPAR [12], as well as PCR [13], have been developed to incorporate complex target-specific probes into the reaction to allow distinction between target-specific and non-specific amplification products; however, this does not prevent the sensitivity of the tests from being jeopardized. The isothermal technology, loop-mediated amplification (LAMP), is an exception that relies on a set of primers, usually between four and six, and a strand displacement polymerase [14]. Although LAMP has exceptional analytical sensitivity and specificity, the method can also suffer from non-specific amplification because of the use of numerous large primers that act as polymerase substrates, resulting in the potential for false-positive results [15], [16].

**Figure 1:**
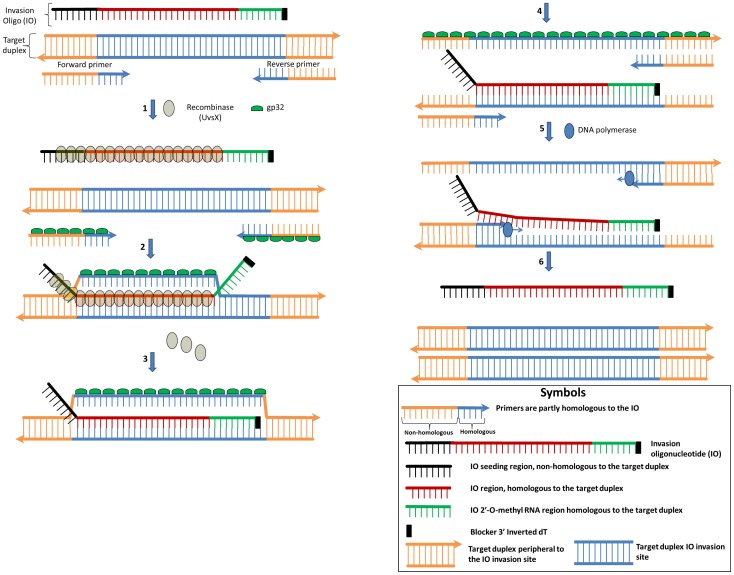
Mechanistic description of the SIBA reaction. All single-stranded elements are coated with gp32, except for the 2′-O-methyl RNA nucleotides. Step 1: UvsX displaces gp32 on the IO and only weakly coats the primers, since they are too short for high affinity binding. Step 2: The IO invades the complementary region of the target duplex which allows partial separation of the target duplex with the downstream end still remaining double-stranded. The out-going strand of the partially separated target duplex is stabilized by gp32. Step 3: UvsX depolymerization allows the 2′-O-methyl RNA region of the IO branch migrates into the duplex. Step 4: Both the upstream and downstream region peripheral to the IO also become short enough to dissociate. Step 5: The strand displacement polymerase is able to extend the dissociated target duplex from the primers. The forward primer displaces the IO during extension of the target template. Step 8: These events lead to the production of two copies of the target duplex. The IO is released to induce further amplification.

Here, we describe a novel isothermal nucleic acid amplification technology, Strand Invasion Based Amplification (SIBA), which is inherently resistant to non-specific amplification and, consequently, sensitive for a single target molecule (Fig. 1). The technology relies on the recombinase-dependent insertion of a single-stranded, invasion oligonucleotide (IO) sequence into a complementary region of a target duplex DNA. The DNA duplex proximal to the IO insertion is disrupted, enabling the binding of target-specific primers, which are not recombinase substrates. A polymerase can then extend the template from the bound primers. Hence, the orchestrated recombinase-dependent insertion of the IO and subsequent polymerase-dependent extension of the primers provide the basis for exponential isothermal amplification. The inclusion of 2′-O-methyl RNA into the sequence of the IO ensures that it is neither a substrate nor a template for the polymerase [17] and it cannot take part in artifactual amplification. Furthermore, since the primers are not recombinase substrates, they are also resistant to the formation of artifacts because they cannot dissociate a duplex in the absence of the IO. These characteristics ensure that amplification occurs only in the presence of the target template. The absence of side reactions ensures that the technology is sensitive to a single copy of a target without the use of target specific probes.

## Materials and Methods

### Materials

All oligonucleotides were purchased from Thermo Fisher Scientific (Germany) or from Integrated DNA Technologies (Belgium), and purified by the manufacturer using reverse phase HPLC (for the primers) and PAGE (for the IO). All oligonucleotides and genomic DNA were diluted and stored in Tris-EDTA (TE) buffer (10 mM Tris-HCL, 0.5 mM EDTA pH 8.0). Details of all the oligonucleotides used in this study are provided in Table S1. All reagents and buffers, including creatine kinase and sucrose phosphorylase, were purchased from Sigma-Aldrich (St. Louis, MO, U.S.A). T4 single-stranded DNA binding protein (gp32) and *Bacillus subtilis* DNA Polymerase I, Large Fragment (BSU) were purchased from New England Biolabs (Ipswich, MA).

### Purification of UvsX and UvsY

UvsX and UvsY were purified as previously described [18], [19]. Briefly, UvsY protein was purified from *Escherichia coli* cells transformed with a temperature inducible plasmid, pTL251W, containing the UvsY coding sequence. Cells were lysed by sonication and the supernatant dialyzed against a buffer containing 20 mM Tris-HCl (pH 7.4), 1 mM EDTA, 5 mM β-mercaptoethanol (BME), 10% (w/v) glycerol and 100 mM NaCl.The dialysate was then subjected to three chromatographic steps using phosphocellulose, ssDNA-cellulose, and hydroxyapatite columns. UvsX protein was purified from *Escherichia coli* cells transformed with the plasmid, pET27b+, which contains the UvsX coding sequence. Cells were then lysed by sonication and the supernatant was dialyzed against a buffer containing 20 mM Tris-HCl (pH 7.4), 5 mM BME, 5 mM EDTA, 50 mM NaCl, and 10% w/v glycerol. The dialysate was then subjected to three chromatographic steps using DEAE cellulose, hydroxyapatite HAP, and Hi Trap Q HP columns. The purity of both UvsY and UvsX was >96%, as judged by SDS-PAGE, and the preparation contained no nuclease activity.

### Bacterial strains and DNA preparation

All bacterial strains used in this study are listed in Table S2. Bacterial strains were cultivated in Brain-heart infusion (BHI) broth at 37°C, except for *Streptococcus agalactiae*, which was cultivated in Todd Hewitt Broth in the presence of 5% CO_2_ at 37°C. Cells were harvested at mid-logarithmic phase and genomic DNA was extracted using the GenElute Bacterial Genomic DNA kit (Sigma). *Salmonella typhimurium* ATCC 14028 was used as a reference strain to assess the sensitivity of the SIBA *Salmonella* assay. A genomic DNA mixture containing 15 non-*Salmonella* strains (*Enterobacter aerogenes* ATCC13048, *Citrobacter sp., Shigella sonnei* ATCC25931, *Shigella flexneri, Streptococcus agalactiae* (B) ATCC12386, *Streptococcus agalactiae* (B) ATCC27956, *Listeria monocytogenes* NCTC11994, *Escherichia coli* ATCC25922, *Enterobacter aerogenes* ATCC15038, *Enterobacter cloacae* 118/1986, *Enterobacter aerogenes* NCTC1006, *Enterobacter spp.* (Paper Mill isolate), *Enterococcus faecalis* ATCC29212, *Citrobacter freundii* ATCC8090, *Klebsiella pneumoniae* ATCC13883 was prepared and used to establish the specificity of the SIBA *Salmonella* assay. The mixture contained equal amounts of genomic DNA copies from each non-*Salmonella* strain.

### SIBA primer and IO configurations

Two functional SIBA assays were used in this study. One was designed to detect an artificial target DNA, while the other was used to detect the InvA gene from *Salmonella* species. The SIBA method required two amplification primers and a IO. The configuration of the primers and IO in relation to the target DNA is shown in Figure S3.

### SIBA reaction conditions

SIBA reactions were performed at 40°C for at least 60 min. Unless otherwise stated, the standard SIBA reaction volume was 20 µl. The components used were 10 mM Tris-acetate (pH 8.0), 10 mM magnesium acetate, 5% DMSO, 5% PEG 1000, 4 mM DTT, 0.5 mM EDTA, 0.1 mg/ml BSA, 150 mM Sucrose, 2 mM ATP, 200 µM dNTPs, 1∶100,000 dilution SYBR Green I, and 60 mM Tris-Phosphocreatine. Additionally, the enzymes used were 250 ng/µl gp32, 140 ng/µl UvsX, 22 ng/µl UvsY, 0.0625 U/µl *Bacillus subtilis* DNA Polymerase I Large Fragment (BSU), 0.0125 U/µl sucrose phosphorylase, and 0.025 U/µl creatine kinase. The concentrations of the primers and IOs used in this study were 200 nM. All reactions were prepared without target DNA or magnesium acetate. The reactions were either started by adding an appropriate amount of target DNA (prepared in magnesium acetate) or magnesium acetate alone.

### Real-time detection, melting curve analysis, and gel analysis of SIBA reactions

Real-time detection of SIBA reactions was performed in a 96-well plate format using the Applied Biosystems ABI 7500 qPCR instrument and accompanying software (version 2.0.6). The instrument was programmed to conduct 60 cycles at 40°C for 60 s with fluorescence readings (SYBR Green I reading by instrument default) collected after each cycle. The specificity of the reaction products was assessed by performing melting curve analysis immediately after the cycles were completed. This was done by heating the reaction rapidly to 95°C for 15 s, followed by a rapid cooling step to 25°C for 60 s. The reactions were then slowly heated from 25°C to 95°C, with fluorescence readings collected at 0.5°C intervals. The reactions were stored at −20°C for further analysis, including non-denaturing polyacrylamide gel electrophoresis (PAGE). For PAGE, a 2 µl aliquot of the reaction mixture was loaded into a 20% TBE gel (Invitrogen, United Kingdom) and electrophoresed at 120 V (constant) for 90 min. Gels were stained with a fluorescent nucleic acid gel stain (GelRed; Biotium, United States) and visualized using a Gel Doc EZ System (BioRad, United Kingdom).

## Results

### Primers do not amplify target DNA sequence independently of the IO

**Figure 2:**
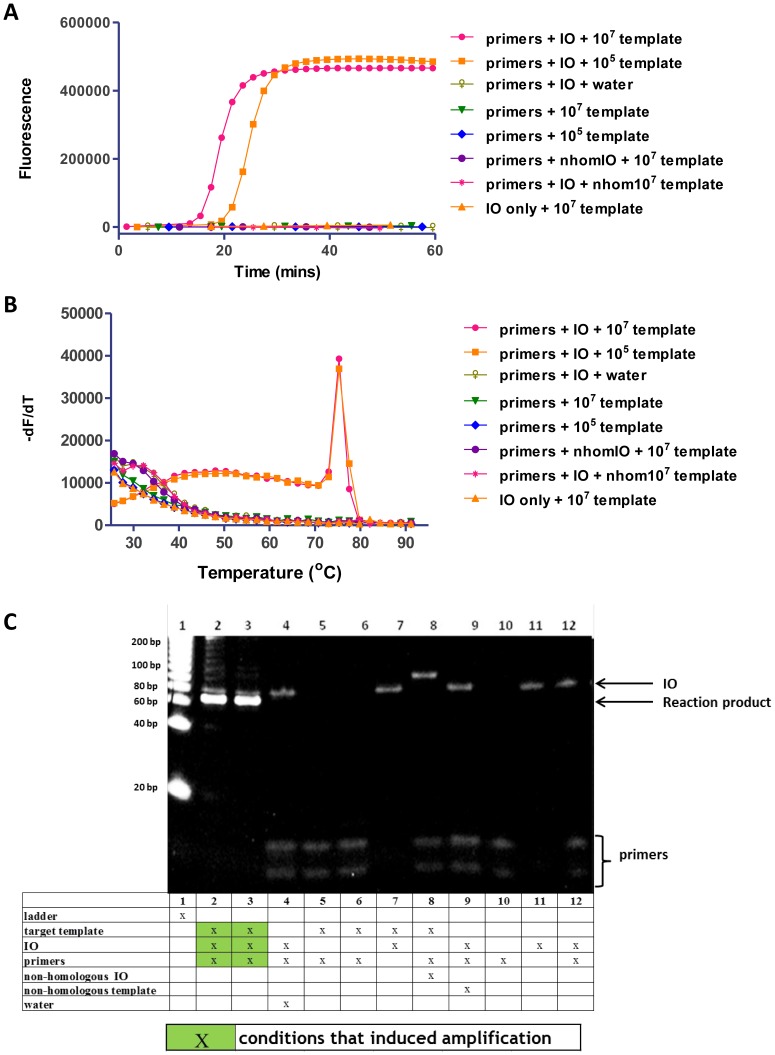
SIBA primers are unable to amplify target DNA independently of the invasion oligonucleotide (IO). (A) Real-time monitoring of amplification using SYBR Green I, (B) melting curve analysis ((-dF (fluorescence)/dT (temperature) versus temperature), and (C) non-denaturing electrophoresis of the corresponding reaction products. Lane 1, BioRad EZ Load 20 bp Molecular Ruler (20–1000 bp); lane 2, primers + IO + template (10^7^ copies); lane 3, primers + IO + template (10^5^ copies); lane 4, primers + IO + water; lane 5, primers + template (10^7^ copies); lane 6, primers + template (10^5^ copies); lane 7, IO + template (10^7^ copies); lane 8, primers + non-homologous IO + template (10^7^ copies); lane 9, primers + IO + non-homologous template (10^7^ copies); lane 10, 200 nM primers in the absence of SIBA reaction reagents; lane 11, 200 nM IO in the absence of SIBA reaction reagents; lane 12, 200 nM primers and 200 nM IO in the absence of SIBA reagents. Lanes 10–12 served as controls for monitoring the presence of oligonucleotides in the reaction products. These were diluted in TBE buffer and run alongside the SIBA reaction products. SB-F21 and SB-R21 are the forward and reverse primers, respectively. The IO used was SB-IO. The homologous target DNA used was SB-template. nhom  =  non-homologous to the target template (SB nhom template) or non-homologous IO (SB nhom IO).

SIBA assay design and reaction scheme is depicted in Figure 1. The methods consists of a modified invasion oligonucleotide (IO) which is a substrate for the recombinase and two terminal primers, a forward and a reverse primer with lengths that are too short to be a substrate for the recombinase. The IO separates the target duplex and peripheral sequence allowing the primers to bind and extend the target duplex. First, we showed that the primers used in SIBA are unable to bind to the target duplex in the absence of a homologous IO; hence, they do not mediate polymerase-mediated extension in this case (Fig. 2). The primers used were below the minimum length required (determined here to be approximately 25–30 nucleotides) for the formation of a recombinase/oligonucleotide complex (pre-synaptic filament), which is necessary for the insertion of a single-stranded DNA molecule into a target duplex. The IO used in SIBA is above this length, enabling its recombinase-dependent insertion into the target duplex [8], [18], [20]. These criteria were confirmed under the SIBA reaction conditions (Figs S1 and S2).

In the present study, the 21 nucleotide SIBA primers (SB-F21 and SB-R21) were incubated with either 10^7^ or 10^5^ copies of target template in the presence or absence of a homologous IO (SB-IO) (Fig. 2A). The IO was 60 nucleotides in length and comprised a non-homologous upstream (seeding) region of 14 nucleotides; this ensured optimal coating of the homologous portion by the recombinase. In addition, the downstream region of the IO comprised 2′-O-methyl RNA nucleotides to ensure that it was not a viable polymerase substrate or template [17]. The assay was performed under SIBA reaction conditions as described in the “Methods” section, and real-time amplification was monitored using SYBR Green I dye. The amplification reaction was detected and expressed as the threshold detection time (dt), which was the time at which the SYBR Green I fluorescent signal exceeded the background signal. Amplification of the target template only occurred in the presence of a homologous IO. The average dt for 10^7^ or 10^5^ target copies was approximately 14 and 18 min, respectively (Fig. 2A). Based on these dt values, the estimated amplification doubling time was calculated to be as low as 35 seconds.

A distinct band of approximately 70 bp corresponding to the amplification product, appeared only in reactions that contained the target template, IO, and primers (Fig. 2C, lanes 2 and 3). This was confirmed by melting curve analysis, which indicated the presence of a single reaction product (Fig. 2B). No amplification was detected in samples without the target template (Fig. 2C, lane 4), demonstrating that non-specific amplification products were absent. A band consistent with the IO remained visible in all reactions containing IO. This confirmed that the IO is not consumed in the SIBA reactions and does not form part of the amplified product, but rather was utilized only for dissociation of the target DNA. To further confirm that the recombinase insertion process was sequence-specific, the homologous IO was replaced with a non-homologous IO (a functional IO homologous to a different target DNA sequence). The non-homologous IO was unable to induce amplification (Fig. 2A, and Fig. 2C lane 8), demonstrating that the insertion process, and consequently the SIBA reaction, is sequence-specific. In addition, neither the homologous IO, nor the primers were able to induce amplification of the target DNA on their own. The primers in the samples in which target-specific amplification occurred were no longer visible on gels (Fig. 2C, lanes 2 and 3). This implies that these primers were totally consumed in the reactions, further demonstrating the efficiency of the reaction. By contrast, the primers were not consumed in reactions performed in the absence of either a homologous DNA template or homologous IO (Fig. 2, lanes 4–6 and 8–10). These findings also demonstrate the absence of non-specific amplification events, such as primer-dimer artifacts. This is likely due to the inability of primer dimers to amplify target sequences, since the primers alone are not recombinase substrates and are therefore unable to invade a duplex. Consequently, amplification is only achievable through homologous insertion by the IO. For a non-target sequence or artifact to be amplifiable, it would need to comprise a sequence region homologous to the IO as well as the primer binding sites. This is a plausible event and was avoided by the inclusion of a 2′-O-methyl RNA region within the IO as described below.

### The importance of the IO 2′-O-methyl RNA region for sensitivity and specificity

The invasion oligonucleotide (IO) sequence used to invade and dissociate the target duplex contained 2′-O-methyl RNA modifications at the 3′-end and was homologous to the target duplex Figure 1. The presence of this modification was critical to the sensitivity and specificity of the reaction. The SIBA reaction is designed such that, after insertion of the DNA region of the IO into a homologous duplex, the 2′-O-methyl RNA region branch migrates further into the duplex, such that approximately 12–16 nucleotides (depending on the sequence) of the duplex remain peripheral to the insertion site. The target duplex peripheral region is defined by the primer region which is non-homologous to the IO. Hence the primers are designed such that their region non-homologous to the IO is approximately 12–16 nucleotides in length.

During the first round of amplification, the primers are able to extend the initial target template added to the reaction (via the action of a polymerase), creating amplicon whose peripheral regions corresponds to the primer length (the length that is non-homologous to the IO). Under the reaction conditions used, target duplex with peripheral regions of this length dissociates (Fig. S7), resulting in complete separation of the target duplex. This allows efficient binding and extension of the target duplex by both primers via the action of a polymerase during each round of amplification. It is, therefore, critical that the length of the amplicon regions peripheral to the IO insertion site is short enough to dissociate under SIBA reaction conditions (Fig. 1). A longer area peripheral to the insertion site results in incomplete dissociation of the target duplex due to the higher melting temperature; thus, very weak or no amplification takes place (Figs S4 and S7). Likewise, if the IO 2′-O-methyl region does not branch migrate, or the primer binding site (i.e the non-homologous region) is too long, amplification will also be very weak or absent (Figs S4 and S7).

SIBA primers are designed to be 16–23 nucleotides long (depending on the sequence) with the 3′-end also homologous to the 2′-O-methyl RNA region of the IO, such that only 12–16 nucleotides remain peripheral (non-homologous to the IO) to the insertion site (Fig. S3). This configuration ensures efficient amplification of the target DNA and minimizes the risk of non-specific amplification. This is because the complete sequence of the IO 2′-O-methyl RNA region is homologous only to the target DNA and cannot be created by the action of the primer/polymerase since this region is not a polymerase substrate. Therefore, a non-specific template will not contain a region homologous to the IO 2′-O-methyl RNA and cannot therefore be efficiently separated by the IO, leading to weak or no amplification (Figs S4, S5, and S7). Here, we demonstrate that the use of this modification successfully ensures the specificity and sensitivity of SIBA reactions.

**Figure 3:**
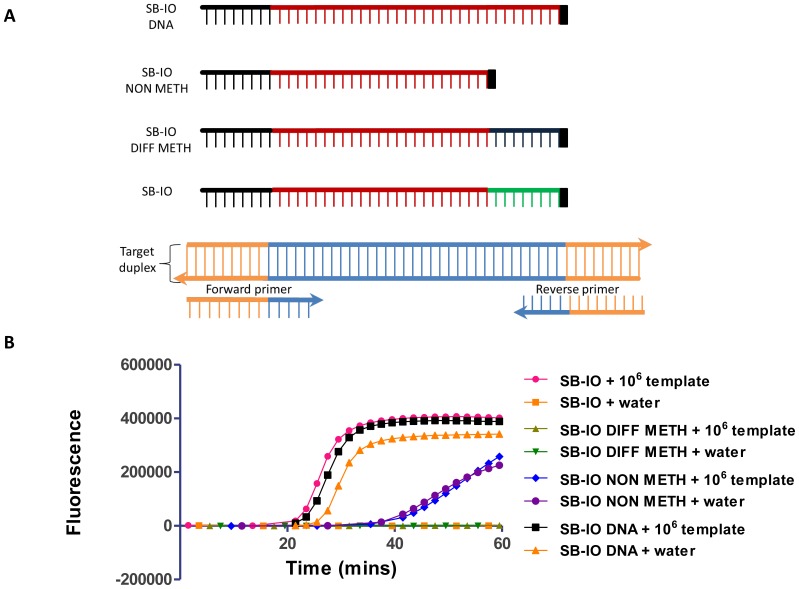
Artifactual amplification is abolished by using an invasion oligonucleotide (IO) with a 2′-O-methyl RNA modification. (A) Configuration of the IO molecules used. (B) Real-time monitoring of SIBA reactions with SYBR Green I using different IOs: (i) IO with a 2′-O-methyl RNA modification and fully homologous to the target duplex, SB-IO; (ii) IO fully homologous to the target duplex, where the 2′-O-methyl RNA modification was replaced with natural DNA nucleotides, SB-IO DNA; (iii) IO with a 2′-O-methyl RNA modification that is not homologous to the target duplex, SB-IO DIFF-METH; and (iv) IO with the 2′-O-methyl RNA modification deleted, SB-IO NON-METH. SB-F21 and SB-R21 were the forward and reverse primers, respectively. The reactions were either performed using 106 target template molecules (SB-template) or in the absence of template.

Four different IOs were used (Fig. 3a): (i) a IO fully homologous to the target duplex with a 2′-O-methyl RNA modification (SB-IO); (ii) a IO with a 2′-O-methyl RNA modification that is not homologous to the target duplex (SB-IO DIFF-METH); (iii) a IO with the 2′-O-methyl RNA modification region deleted (SB-IO NON-METH) and (iv) a IO fully homologous to the target duplex with the 2′-O-methyl RNA modification replaced by natural DNA nucleotides (SB-IO DNA). These IOs were used to amplify the target template in the presence of the target-specific primers. The reaction was also conducted in the absence of the target template to evaluate its specificity. The results are shown in Figure 3B.

The use of a fully homologous IO with a 2′-O-methyl RNA modification (SB-IO, Fig. 3B) led to the amplification of the target template without generating any non-specific amplification products and this was the same IO used in Figure 2. When a IO with a non-homologous 2′-O-methyl RNA region was used, no amplification was observed either in the presence or absence of template (SB-IO DIFF-METH, Fig. 3B). Such a IO (with a non-homologous 2′-O-methyl RNA region) is unable to branch migrate into the target duplex; therefore, the residual duplex, even after insertion of the IO DNA region, remains too long to dissociate. This results in only partial dissociation of the target duplex, compromising the binding site for the amplification primers (Figs S5 and S7). Interestingly, IOs that completely lacked a 2′-O-methyl RNA region (SB-IO NON-METH) tended to produce detectable, albeit weak, amplification, as well as some non-specific artifacts. Despite the 3′-terminating nucleotide base (reversed dT), it is plausible that the reaction is not complete, resulting in some extendible IOs and the production of artifacts. Replacement of the 2′-O-methyl RNA modification with natural DNA nucleotides (SB-IO DNA) also permitted amplification but led mainly to the generation of non-specific amplification products. Since the reverse primer is partly complementary to the 3′-end of the IO, it is able to bind to and enable the extension of the IO to create an amplifiable duplex in either the presence or absence of a target template. This emphasizes the importance of a IO that is not a substrate for polymerase.

### The sensitivity of SIBA extends to a single point mutation

**Figure 4:**
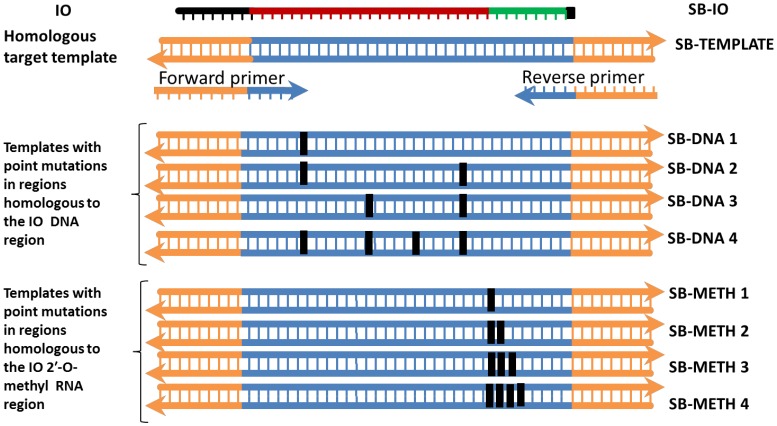
Configuration of templates used for evaluating the sensitivity of SIBA for point mutation(s). Eight additional templates harboring different numbers of point mutation(s) 1–4 were synthesized. The point mutation(s) were either located in regions homologous to the IO DNA or the IO 2′-O-methyl RNA region (SB-IO). The black box on the templates indicates the location of the point mutation (s).

Complete dissociation of the target duplex upon invasion by the IO is required for efficient amplification. A template whose sequence is not fully homologous to both the primers and the IO may amplify poorly or not at all. We sought to elucidate the impact of 1–4 base point mutations on the recombinase-dependent insertion of the IO and the efficiency of the subsequent amplification. DNA templates harboring point mutations of 1–4 bases were synthesized (Fig 4). The mutations were either located in regions homologous to the IO (SB-IO) DNA or the IO 2′-O-methyl RNA region. None were located in the primer binding sites; hence, all templates containing point mutation(s) remained fully homologous to the same primers (SB-F21 and SB-R21). Therefore, the specificity shown in this experiment relates only to the recombinase-dependent insertion of the IO. The assay was performed using 10^4^ copies of the templates and real-time monitoring of amplification was achieved using SYBR Green I. The results are expressed as the delay in the threshold detection time (dt) i.e., mean Δdt =  mean dt of a template containing point mutation(s) – mean dt of the fully homologous target template (SB-template) (Table 1).

**Table 1:** Sensitivity of SIBA extension to point mutations.

	No. of point mutations			
	1	2	3	4
**Location of point mutation(s) within the amplification template**	**Δdt (Minutes)**			
Homologous to the IO DNA region	**SB-DNA1**	**SB-DNA2**	**SB-DNA3**	**SB-DNA4**
	7.4	24.0	ND	ND
Homologous to the IO 2′-O-methyl RNA region	**SB-MET1**	**SB-MET2**	**SB-MET3**	**SB-MET4**
	11.7	ND	ND	ND

The SIBA reaction was performed with either a fully homologous target template (SB-template) or with templates containing 1–4 base point mutation(s). The results are expressed as the delay in the threshold detection time (dt), i.e., the average Δdt =  average dt of a template containing point mutation(s) minus the average dt of the fully homologous target template (SB-template). ND denotes no detectable amplification of a template. SB-F21 and SB-R21 were the upstream and downstream primers, respectively. The invasion oligonucleotide (IO) used was SB-IO.

A single point mutation in the template, located either in the region homologous to the IO DNA or the 2′-O-methyl RNA region, led to a dramatic decrease in amplification efficiency, reflected by an increase in the threshold detection time (dt). The effect was slightly more accentuated when the single point mutation was located in the region homologous to the 2′-O-methyl RNA region rather than in the DNA region (Δdt = 11.7 and 7.4 minutes respectively). The rate of IO 2′-O-methyl RNA branch migration is likely to become less efficient when it is not fully homologous to the template, a feature essential for efficient amplification (Fig. 3). With respect to a point mutation in the region homologous to the IO DNA region, it is likely that the recombinase can tolerate at least one incorrect base without preventing invasion. This could be an advantage in situations in which the detection of closely-related targets that differ by one or a few bases is required. Furthermore, it is plausible that the polymerase error rate would eventually induce a mutation that would revert the amplification product to the correct template sequence. The dt increased further as the number of point mutations increased. A template with double point mutations at regions homologous to the IO DNA region showed a further delay in dt (Δdt = 24.0 minutes). Interestingly, a template containing double point mutations at regions homologous to the IO 2′-O-methyl region did not amplify; therefore, the change in dt could not be determined. No amplification was observed in reactions containing templates harboring three or four point mutations located in either the region homologous to the IO DNA or in the 2′-O-methyl RNA site. Discriminating a single point mutation is only achievable via real-time detection, since DNA targets that differ by a single point mutation are still detected in SIBA reactions even though they differ in detection time. Discriminating two or more point mutations maybe more reliable since double point mutations located at regions homologous to the IO 2′-O-methyl region can abolish amplification.

The result could also serve as a tool for designing SIBA assays that require inclusivity or exclusivity of closely related targets. When detecting all closely related target DNA that differs by 1 or 2 nucleotides is of interest, then SIBA assays could be designed such that the location of the point mutation (s) on target DNA is homologous to the DNA region of the IO. On the other hand, if detecting a particular target DNA with the exclusion of other closely related targets that differ by 2 or more nucleotides is of interest, then SIBA assays could be designed such that the location of the point mutation (s) on target DNA is homologous to the IO 2′-O-methyl RNA region. This is due to the fact that the 2′-O-methyl RNA region of the IO appears to be more sensitive to point mutation than the DNA region on the IO.

### Application of SIBA to detect *Salmonella* at single molecule sensitivity without amplifying closely-related species

**Figure 5:**
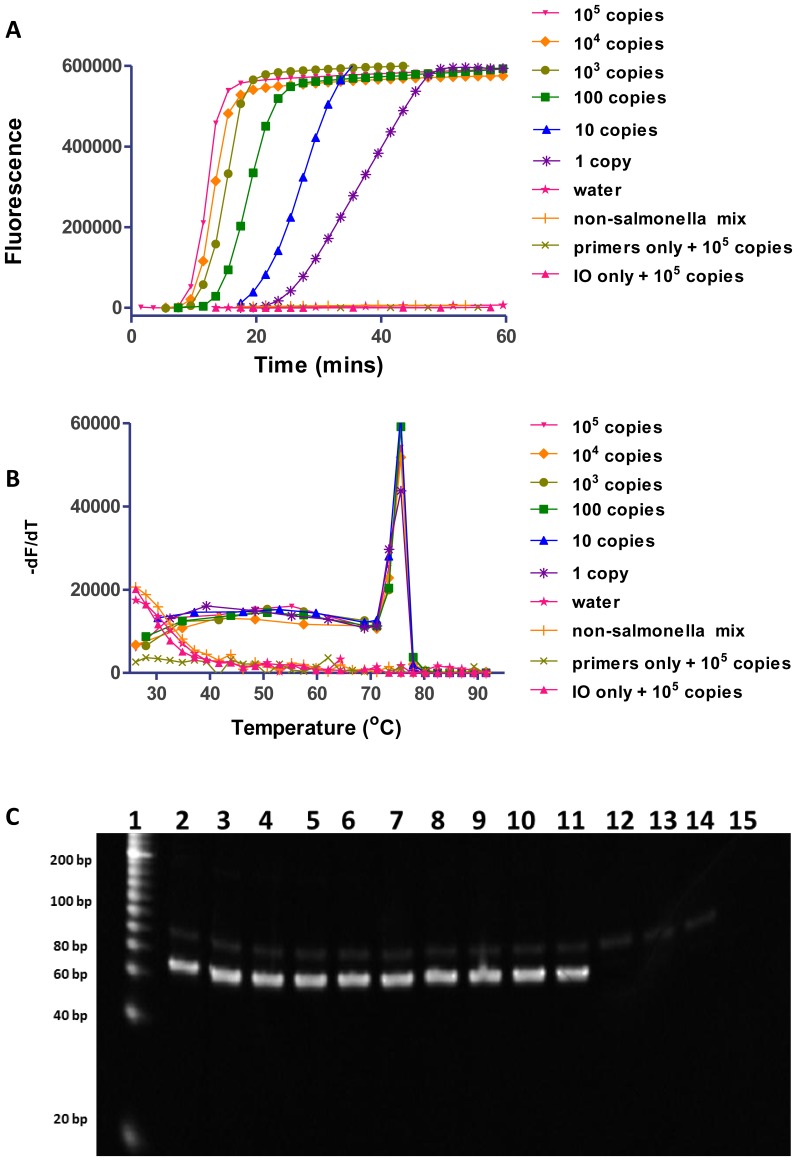
Determination of SIBA sensitivity for *Salmonella* genomic DNA. (A) Real-time monitoring of DNA amplification using SYBR Green I, (B) melting curve analysis, ((-dF (fluorescence)/dT (temperature) versus temperature) and (C) electrophoresis of the corresponding reaction products. Lane 1, BioRad EZ Load 20 bp Molecular Ruler (20–1000 bp); lanes 2–11 primers + IO + 10^5^, 10^4^, 10^3^, 100, 50, 20, 10, 5, 2, or 1 copy of *Salmonella* genomic DNA, respectively; lane 12, primers + IO + water; lane 13, primers + IO + a mixture of non-*Salmonella* spp. genomic DNA (each spp. contains 1000 copies per reaction); lane 14, IO + 10^5^ copies *Salmonella* genomic DNA; lane 15, primers + 10^5^ copies *Salmonella* genomic DNA; 50, 20, 5 and 2 copies were omitted from Figure 5a and b for the sake of clarity. SM-F18 and SM-R16 were the forward and reverse primers, respectively. The IO used was SM-IO.

Here, we demonstrated the sensitivity and specificity of SIBA technology by amplifying the InvA gene from *Salmonella* genomic DNA (Fig. 5). Primers and the IO were designed as described in the SIBA method (Fig. S3). When *Salmonell*a genomic DNA is used as the initial target template, its peripheral region is too long to dissociate during the first round of amplification (Fig. S7). However, in the subsequent rounds of amplification, the peripheral region created by the primers after the initial extension of the target DNA becomes short enough to dissociate. A delay of around 5 minutes is usually observed when non-denatured genomic DNA is used as the initial target template compared to denatured genomic DNA (Fig. S8). We were able to amplify sequences from the InvA gene efficiently using complex bacterial genomic DNA as the template (Fig. 5A–C).

The sensitivity of the test was evaluated in at least three independent experiments by serially diluting *Salmonella* genomic DNA from 10^5^ copies to one copy in quadruplicate (Fig. 5). *Salmonella* genomic DNA was incubated with 90% DMSO at room temperature for 5 minutes and diluted with TE (10 mM Tris–HCL, 0.5 mM EDTA pH 8.0) buffer devoid of DMSO before being added to the reaction. This short pre-incubation of the genomic DNA with 90% DMSO minimized the short delay associated with the first round of amplification (Fig. S8) [21]. SIBA detected ten copies of genomic DNA in the samples (Fig. 5A) within 20 minutes of starting the reaction. In samples containing five, two, or one copy of *Salmonella* genomic DNA, only ¾, ½, and ¼ of the samples, respectively, were amplified. This is likely to reflect the inconsistent presence of DNA at such low copy numbers. SIBA was also able to detect a very low number of copies of other target DNAs (Fig. S6). Similar sensitivity was observed in other SIBA assays. A few isothermal technologies show similar or slightly quicker detection times than SIBA; however, these methods are unable to efficiently amplify such low copy number targets robustly due to competition between non-specific side reactions and target-dependent amplification [22], [23]. Also, they may require complex probes for detection [8].

The amount of product formed from the different initial amounts of *Salmonella* genomic DNA was similar, and the no-template controls (NTC) did not produce any detectable signal, confirming that the method does not support artifactual amplification. The specificity of the reaction product was further confirmed by melting curve analysis and electrophoresis (Fig. 5B–C). The specificity of SIBA was investigated by performing reactions in the presence of mixed excess genomic DNA from 15 non-*Salmonella* species, which are non-homologous to the target gene sequence (1000 copies of genomic DNA per reaction was added per species). No amplification was detected using this non-*Salmonella* mixture. This is not surprising since SIBA is sensitive to point mutations and can be used to distinguish between closely-related strains (Fig. 4 and Table 1).

## Discussion

This study describes the development of a novel isothermal nucleic acid amplification method with high analytical sensitivity and specificity. Despite the introduction of numerous isothermal nucleic acid technologies, their uptake in the field of molecular diagnostics has been limited. One reason for this is that these methods can be prone to generating non-specific amplification products (thereby increasing the risk of false-positive results) [15], [16] unless complex target-specific probes are used to distinguish between target-specific and non-target-specific amplification [8]. Nevertheless, these non-specific side reactions consume assay components, leading to reduced sensitivity and the potential for false-negative results; therefore, meticulous handling may be required [5], [7]. SIBA eliminates non-specific amplification because, in contrast to other systems such as RPA and HDA, SIBA primers cannot bind to or allow the extension of a template in the absence of IO [8]. In addition, any artifacts produced during the SIBA reaction are amplified very inefficiently compared with the target-dependent reaction.

The SIBA reaction relies on a complex sequence of events and, consequently, avoids the formation of amplifiable artifacts. An oligonucleotide (IO) invades a duplex DNA via the action of a recombinase. As a result, the 2′-O-methyl RNA region (which forms the 3′-terminus of the IO molecule) branch migrates further into the duplex. This leaves only a short intact region of the duplex, which then completely dissociates to enable the amplification primers to bind and be extended by the polymerase. The primers cannot independently bind the target duplex in the absence of the IO as they are not recombinase substrates. The IO can neither be amplified nor extend the target duplex since it is not a substrate for polymerase.

It is plausible that, on occasion, a primer could copy onto the IO and then, through a process of strand switching, further copy onto another primer. This would induce an amplifiable artifact (Fig. S5). Such a configuration would rely on the primers being short enough to leave only a small peripheral duplex should they be incorporated into an artifact, enabling complete dissociation of the duplex during amplification. The challenge here was to design a system in which the primers would be too long for amplification to occur when incorporated into an artifact, but short enough to enable amplification of the target of interest. The solution was achieved using primers bearing 3′-ends homologous to the IO. In this configuration, the primer leaves only a short peripheral region when the target DNA is amplified, but a long peripheral region when incorporated into an artifact. Although the 3′-end of the reverse primer is complementary to the IO, it lies within the 2′-O-methyl RNA region, which is not a template for polymerase [17]; therefore, there is no risk that the primer will extend this region. Even a single base change in the 2′-O-methyl region inhibited its ability to branch migrate. Consequently, SIBA is not only highly sensitive due to its resistance to non-target-dependent amplification, but is also highly specific (Fig. 4, Table 1, and Fig. S7). It is difficult to theoretically predict what the ideal melting temperatures of these primers should be since this is strongly influenced by the SIBA buffer conditions, particularly the presence of gp32. However, we found that primers up to 23 nucleotides long, with around 14 nucleotides non-homologous to the IO, are ideal for SIBA. One strategy for achieving the ideal primer length is to design several primers (e.g., four forward and four reverse primers) that differ in their lengths of homologous and non-homologous regions relative to the IO insert. These primers are then tested in various combinations in the presence or absence of the target DNA and IO. The ideal primer combination can then be chosen based on their ability to efficiently amplify the target DNA.

The SIBA reaction includes a recombinase, recombinase cofactors, and an energy generating system, as well as the nucleic acid components and polymerase. Additionally, a crowding agent and sucrose phosphorylase are included. UvsX has a very rapid turnover of ATP, which indicates that it is an efficient recombinase, but rapidly generates inorganic phosphate, which is highly inhibitory [20]. The inclusion of sucrose and sucrose phosphorylase remove inorganic phosphate without requiring further high energy cofactors [24]. Polyethylene glycol (PEG), which enhances the efficiency of recombinase systems, is also beneficial for SIBA [25]. It is also important to ensure that the oligonucleotides used in SIBA are of a high quality and (preferably) HPLC purified, since a truncated IO might still be able to invade the target but will lead to poor amplification due to its inability to dissociate the duplex. This will result in competition with the competent portion of the IO preparation. The optimum concentrations of the recombinase, UvsX, and accessory proteins used in SIBA differ from one target analyte to another. This may be because UvsX shows differing affinity for different DNA molecules, which is dependent on length as well as composition. For example, UvsX binds preferentially to pyrimidines [20]. In addition, at the high concentrations of UvsX used in SIBA, UvsY was not necessary and can be inhibitory, which is consistent with previous findings [26]. The ability of oligonucleotides ≥25-mer to support invasion in the presence of recombinase is consistent with previously reported data [8], [20].

SIBA and RPA[8] depend on the application of the recombinase UvsX during the amplification process. The primers used in RPA are substrates for the recombinase which induces their insertion into the target duplex. In contrast, the primers used in SIBA are short and consequently are not recombinase substrates. They were shown to be unable to invade the target duplex on their own. In SIBA, a non-extendible invasion oligonucleotide (IO) is the recombinase substrate and is inserted between the two primer binding sites. This results in the separation of the duplex peripheral to the IO insertion site enabling the primers to bind and extend the target DNA via the action of a polymerase. The IO is neither consumed nor takes part in the extension of the target DNA. The advantage of this process is that, due to the incompetence of the primers alone in SIBA as well as the non-extendible nature of the IO, primer artefacts are abolished. These characteristics ensure that SIBA is able to detect a single copy of target DNA without generating non-specific amplicons. Such sensitivity is not usually achievable with isothermal methods, since the generation of non-specific amplification products is prominent at low target copy numbers or in the absence of a target DNA [5]. For example, amplification of a DNA target from *Treponema denticola* using HDA resulted in a decreasing amount of product as the initial copy number used decreased [7]. Furthermore, non-specific amplification could be seen in the no-template control, presumably as a result of primer-dimer artifacts. Several of these isothermal methods, such as RPA, use complex target-specific probes in an attempt to distinguish between target and non-specific amplification. However, because the assay components are consumed, these non-specific reactions may amplify at rates that are faster than the rate of target DNA amplification, leading to a loss of sensitivity. In SIBA, such problems are absent since it is designed in such a way that only target-specific DNA can be amplified efficiently. SIBA can be used to reliably detect low copy numbers of pathogens, as demonstrated by the detection of low copy numbers of *Salmonella* DNA, and is suitable for both routine centralized as well as decentralized testing since the method can be performed with low-cost instruments. Low cost devices have been developed for fluorescent detection of common isothermal DNA amplification methods such as RPA and LAMP. These can also be used to monitor the SIBA reaction product as well as for assessing reaction product specificity. A microplate reader or fluorometers with temperature control function are also suitable for detecting SIBA reaction products. SIBA reactions can also be performed using an incubator set at 40°C. The reaction product can then be detected by gel electrophoresis if needed.

## Supporting Information

Figure S1 **DNA pairing strand exchange assay using labeled duplexes of using different lengths.**
(PDF)

Figure S2 **Primers < 21 nt in length are unable to amplify the target template.** (A) Configuration of the template and primers used. (B) Real-time monitoring of SIBA reaction amplification using SYBR Green I. (C) Gel electrophoresis of the corresponding SIBA reaction products. Template (10^7^ copies) or nucleic acid-free water (NFW) were used in the absence of IO. Lane 1, BioRad EZ Load 20 bp Molecular Ruler (20–1000 bp); lane 2, SB-F21/SB-R21 + template; lane 3, SB-F18/SB-R18 + template; lane 4, SB-F16/SB-R16 + template; lane 5, SB-F14/SB-R14 + template; lane 6, SB-F30/SB-R30 + template; lane 7, SB-F35/SB-R35 + template; lane 8, SB-F30/SB-R30 + water; lane 9, SB-F35/SB-R35 + water. SB-TEMPLATE LONG (10^7^ copies) was used.
(PDF)

Figure S3 **Schematic representation of the SIBA primers and invasion oligonucleotide (IO) used amply an (A) artificial system or (B) the Salmonella InvA gene.** The underlined region of the primer is the sequence that is homologous to the IO. The green region of the IO (marked in italics) is modified with 2′-O-methyl RNA. The bold sequence within the IO represents the region that is non-homologous to the target DNA.
(PDF)

Figure S4 **Primer length constraints.** (A) Configuration of the forward and reverse primers containing non-homologous regions of different lengths. Blue indicates the region of the target template that is homologous to the IO. The black line on the IO indicates the non-homologous seeding region. The green lines on the IO indicate the 2′-O-methyl RNA region and the black square indicates the inverted dT modification. (B) Real-time monitoring of SIBA reactions with SYBR Green I using various combinations of forward and reverse primers. SB-IO was the IO used in this study. The reactions were performed with 10^4^ copies of SB-long target template.
(PDF)

Figure S5 **The sensitivity of SIBA is improved by using long primers that are partially homologous to the IO.** (A) Configuration of the forward and reverse primers used. The orange line on the primers indicates the region that is non-homologous to the IO. (B) The SIBA reaction was performed using either 10^4^ copies of target DNA (SB-template) or water. Amplification was monitored in real-time using SYBR Green I. The forward primer was either short (non-homologous to the IO; SB-F14) or long (3′-end homologous to the IO; SB-F21). The reverse primer was SB-R21. The IO used was SB-IO. **Fig. S5c: Non-specific artifacts are not amplified in the SIBA reaction.** Step 1: Primers can non-specifically copy onto the IO; however, this will not include the 2′-O-methyl RNA region since it is not a substrate for polymerase. Step 2: Through a process of strand switching, the product may further copy onto another primer. Step 3: The product formed can have a region homologous to the IO insertion but not to the 2′-O-methyl RNA region. Step 4: The region peripheral to the IO will not dissociate since it is too long to dissociate.
(PDF)

Figure S6 **Sensitivity of the SIBA assay.** (A) Amplification of target DNA serially diluted from 10^6^ copies to a single copy (SB-template) per sample. Amplification was monitored in real-time using SYBR Green I. (B) Melting curve analysis of the corresponding reaction products ((-dF (fluorescence)/dT (temperature) versus temperature). SB-F21 and SB-R21 were the forward and reverse primers, respectively. The IO used was SB-IO.
(PDF)

Figure S7 **DNA pairing strand exchange assay of a labeled duplex using different IO configurations.** (i) IO fully homologous to the target duplex with a 2′-O-methyl RNA modification, IO-METH. (ii) IO fully homologous to the target duplex with the 2′-O-methyl RNA modification replaced with natural DNA nucleotides, IO-DNA. (iii) IO with a 2′-O-methyl RNA modification that is not homologous to the target duplex, IO DIFF-METH. (iv) IO with the 2′-O-methyl RNA modification deleted, IO-SHORT. Two duplexes, in which the lengths of the downstream region peripheral to the IO differed (14 bp and 25 bp), were used.
(PDF)

Figure S8 **Minimal lag time associated with the first round of amplification.** The SIBA *Salmonella* assay was performed in the presence or absence of a restriction enzyme, RsaI either with *Salmonella* genomic DNA or pre-denatured *Salmonella* genomic DNA (using DMSO). Amplification was monitored using SYBR Green I.
(PDF)

Table S1 **Oligonucleotides used in this study.**
(PDF)

Table S2 **Bacterial strains used in this study.**
(PDF)
