# Supplementary material for: Strand Invasion Based Amplification (SIBA®): A Novel Isothermal DNA Amplification Technology Demonstrating High Specificity and Sensitivity for a Single Molecule of Target Analyte
Source: PLoS One. 2014 Nov 24;9(11):e112656. doi: 10.1371/journal.pone.0112656 (PMC4242538; doi:10.1371/journal.pone.0112656)
Supplement: Figure S1 — DNA pairing strand exchange assay using labeled duplexes of using different lengths. (PDF) [file pone.0112656.s001.pdf]

Supporting information S1

Oligonucleotides below 25 nucleotides in length are unable to invade a duplex.

The minimum oligonucleotide length required for recombinase mediated invasion of a duplex DNA was elucidated. DNA strand exchange assay was performed using labeled duplex DNA whose 3’ and 5’ ends were labeled with tetramethylrhodamine (TAMRA) and fluorescein (FAM), respectively, to form a fluorescence resonance energy transfer (FRET) system [1]. Labeled duplexes of different lengths were created by incubating a 50-mer oligonucleotide (50 nM invasion template) labeled with FAM at the 5'-end with complementary oligonucleotides (100 nM) of different lengths (25-, 35-, 40-, and 49-mers) labeled with TAMRA at the 3'-end. All duplexes formed were aligned in such a way that the FRET remained unchanged.

The ability of six unlabeled complementary oligonucleotides (100 nM) of different lengths (16–49-mer) to invade the labeled duplex strand was then determined. The length of the unlabeled oligonucleotides (25–49 nucleotides) was equal to that of the duplex used in the corresponding invasion assay. For unlabeled complementary oligonucleotides ≤ 21 nucleotides, the length of the labeled duplex used was 25 bp. This was because no stable duplexes less than 25 bp were produced under SIBA reaction conditions.

Strand exchange was determined by the increase in fluorescein signal arising from the displacement of the TAMRA-labeled strand by the unlabeled oligonucleotides. The experiment was performed under standard SIBA reaction conditions (see Materials and Methods), excluding polymerase and dNTPs. Endpoint fluorescence after 20 min incubation at 40°C was measured using the Agilent MX pro device (Agilent Technologies, Inc., USA). The results are presented as the percentage of invasion by normalizing the fluorescein signal produced after the strand exchange reaction to that of a corresponding heat denatured duplex (95°C, 10 min). Here, we showed that oligonucleotides below 25 nucleotides in length are unable to support recombinase mediated DNA strand exchange (Fig. S1). This is consistent with previous reports showing that UvsX cannot form stable filaments or initiate strand exchange with short oligonucleotides [2,3]. Primers used in SIBA are below this minimum length and are therefore unable to invade a duplex. Conversely, the IO used in SIBA must be above 25 nucleotides length in order to support recombinase mediated strand invasion.

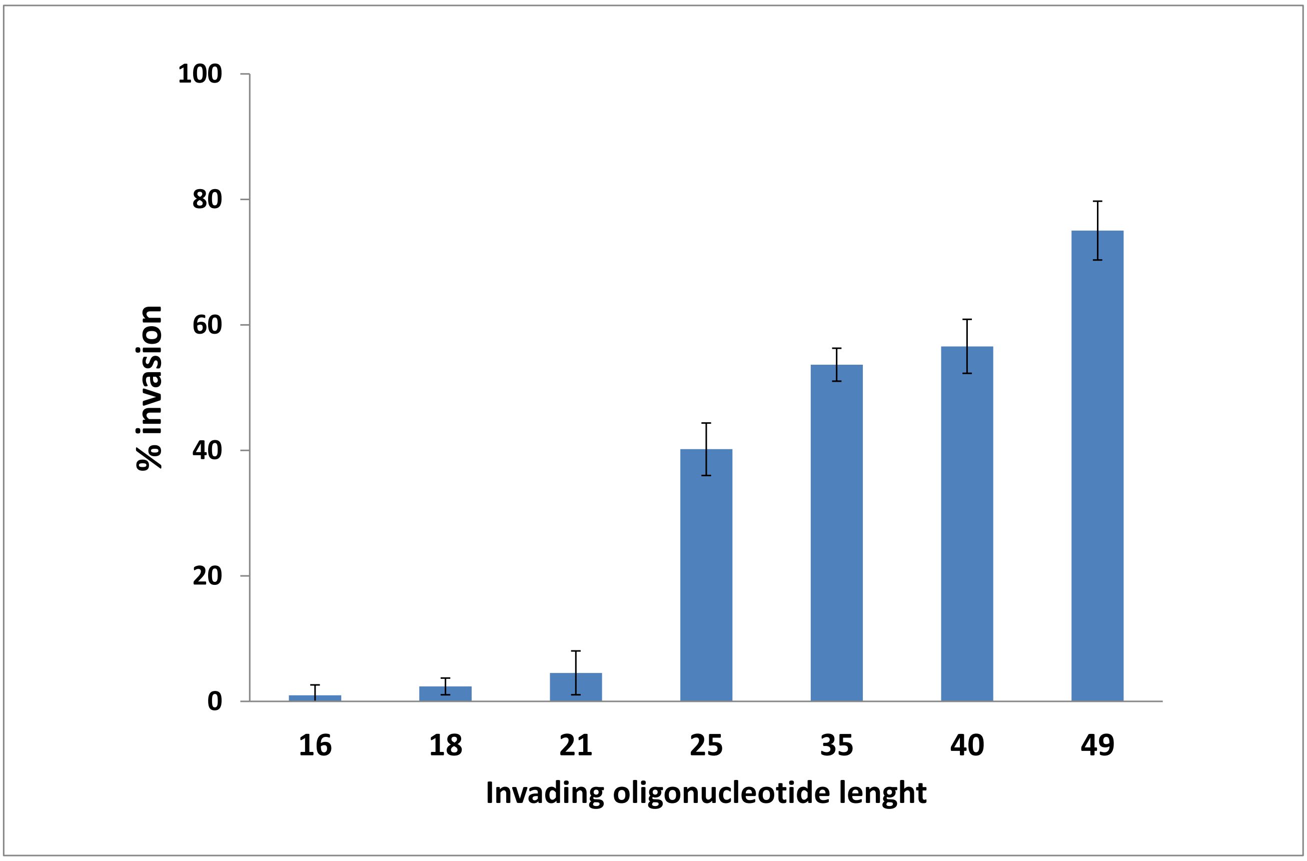

Figure S1. DNA pairing strand exchange assay using labeled duplexes of using different lengths

References

1. Bazemore LR, Takahashi M, Radding CM (1997) Kinetic Analysis of Pairing and Strand Exchange Catalyzed by RecA: DETECTION BY FLUORESCENCE ENERGY TRANSFER. Journal of Biological Chemistry 272: 14672-14682.

2. Formosa T, Alberts BM (1986) Purification and characterization of the T4 bacteriophage uvsX protein. Journal of Biological Chemistry 261: 6107-6118.

3. Gamper HB, Nulf CJ, Corey DR, Kmiec EB (2003) The Synaptic Complex of RecA Protein Participates in Hybridization and Inverse Strand Exchange Reactions†. Biochemistry 42: 2643-2655.
