# Supplementary material for: Strand Invasion Based Amplification (SIBA®): A Novel Isothermal DNA Amplification Technology Demonstrating High Specificity and Sensitivity for a Single Molecule of Target Analyte
Source: PLoS One. 2014 Nov 24;9(11):e112656. doi: 10.1371/journal.pone.0112656 (PMC4242538; doi:10.1371/journal.pone.0112656)
Supplement: Figure S2 — Primers < 21 nt in length are unable to amplify the target template. (A) Configuration of the template and primers used. (B) Real-time monitoring of SIBA reaction amplification using SYBR Green I. (C) Gel electrophoresis of the corresponding SIBA reaction products. Template (107 copies) or nucleic acid-free water (NFW) were used in the absence of IO. Lane 1, BioRad EZ Load 20 bp Molecular Ruler (20–1000 bp); lane 2, SB-F21/SB-R21 + template; lane 3, SB-F18/SB-R18 + template; lane 4, SB-F16/SB-R16 + template; lane 5, SB-F14/SB-R14 + template; lane 6, SB-F30/SB-R30 + template; lane 7, SB-F35/SB-R35 + template; lane 8, SB-F30/SB-R30 + water; lane 9, SB-F35/SB-R35 + water. SB-TEMPLATE LONG (107 copies) was used. (PDF) [file pone.0112656.s002.pdf]

Supporting information S2

Primers used in SIBA (< 21 nucleotides in length) are unable to amplify target template

The primer lengths required for recombinase dependent amplification of a target DNA was investigated. Target specific primers pairs (i.e. forward and reverse primer) of different lengths (14-35 nucleotides) were incubated with a target DNA in the absence of the IO, under standard SIBA reaction conditions described in the "Materials and Methods" section. Figure S2A shows the target DNA and primer configurations/lengths used in this experiment. Primers (200 nM) were incubated both in the presence and absence of 10^7 copies of target DNA . The results can be seen in Figure S2B. Primers ≤ 21 nucleotides in length did not produce any detectable increase in SYBR Green I signal either in the presence or absence of the target DNA This suggests that primers ≤ 21 nucleotides (nt) in length are unable to amplify the target template. We reasoned that since they are below the minimum length required for recombinase dependent invasion of a duplex (Fig. S1), they are also unable to extend the target DNA via the action of a polymerase.

.

Primers ≥ 30 nt in length yielded a detectable increase in the SYBR Green I signal, suggesting that amplification of the target template occurred. Primers ≥ 30 nucleotides in length are able to invade the target duplex and as such the extension of the target DNA can take place via the action of a polymerase (Fig. S1). The orchestrated recombinase dependent primer invasion of the target duplex and subsequent polymerase-dependent extension can lead to an exponential amplification. This is consistent with previous studies demonstrating that primer recombinase-dependent amplification requires primers greater than 27 nt in length. This is the basis for a previously described isothermal amplification method, Recombinase Polymerase Amplification (RPA) [1]. However, as seen in Figure 2B and 2C, non-target-dependent amplification was also observed for primers ≥ 30 nucleotides in length. The electrophoresis results suggested that only primers of 35 nucleotides yielded the correct amplification product (Fig. S2C). The non-target- amplification seen with primers ≥ 30 nt could be due to primer dimers. The resulting aberrant template is able to exponentially amplify, since primers ≥ 30 nt are able invade and extend the template via the action of a recombinase and a polymerase respectively. In contrast, aberrant template resulting from primers ≤ 21 nt in length, are unable to exponentially amplify since they not recombinase substrates and are

therefore unable to invade a duplex. Primers used in SIBA ≤ 21 nt in length and a likely to be more resistant to non-specific amplification since they are not substrate for a recombinase.

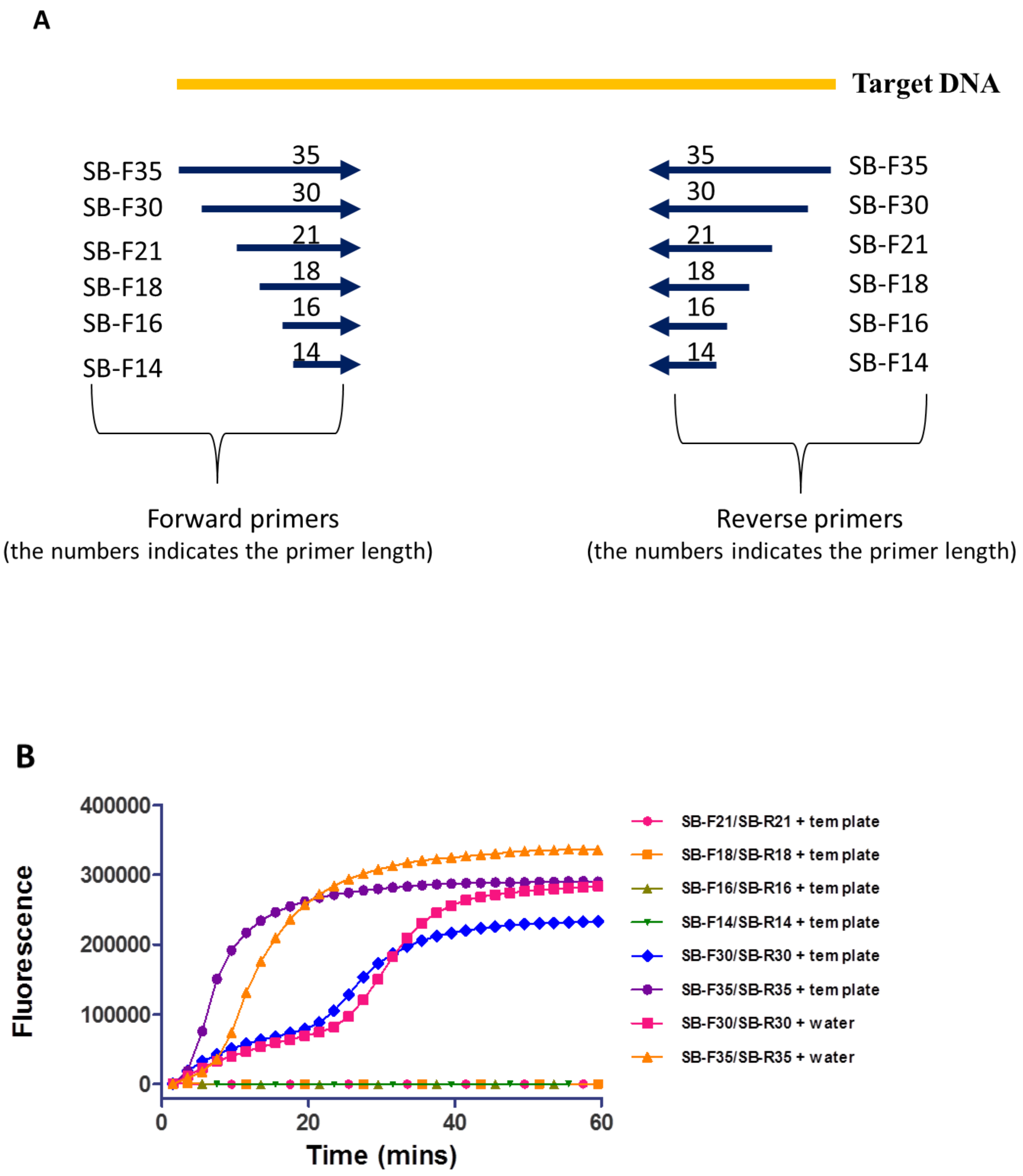

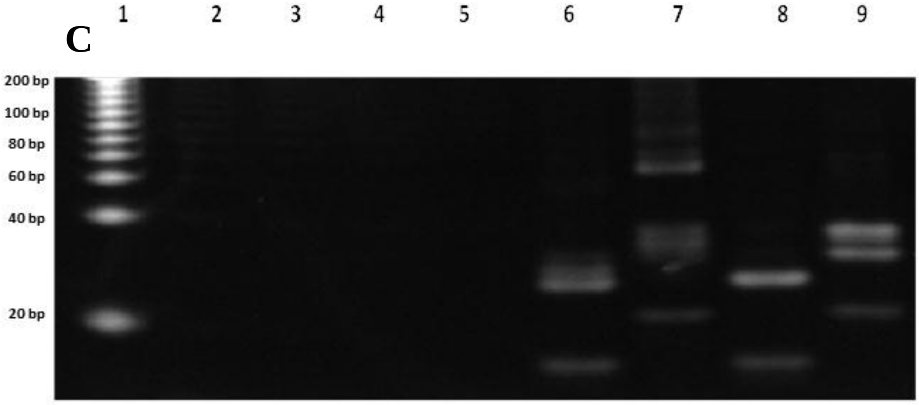

Figure S2. Primers < 21 nt in length are unable to amplify the target template. (A) Configuration of the template and primers used. (B) Real-time monitoring of SIBA reaction amplification using SYBR Green I. (C) Gel electrophoresis of the corresponding SIBA reaction products. Template (10^7 copies) or nucleic acid-free water (NFW) were used in the absence of IO. Lane 1, BioRad EZ Load 20 bp Molecular Ruler (20–1000 bp); lane 2, SB-F21/SB-R21 + template; lane 3, SB-F18/SB-R18 + template; lane 4, SB-F16/SB-R16 + template; lane 5, SB-F14/SB-R14 + template; lane 6, SB-F30/SB-R30 + template; lane 7, SB-F35/SB-R35 + template; lane 8, SB-F30/SB-R30 + water; lane 9, SB-F35/SB-R35 + water. SB-TEMPLATE LONG (10^7 copies) was used.

References

1. Piepenburg O, Williams CH, Stemple DL, Armes NA (2006) DNA Detection Using Recombination Proteins. PLoS Biol 4: e204.
