# Supplementary material for: Strand Invasion Based Amplification (SIBA®): A Novel Isothermal DNA Amplification Technology Demonstrating High Specificity and Sensitivity for a Single Molecule of Target Analyte
Source: PLoS One. 2014 Nov 24;9(11):e112656. doi: 10.1371/journal.pone.0112656 (PMC4242538; doi:10.1371/journal.pone.0112656)
Supplement: Figure S3 — Schematic representation of the SIBA primers and invasion oligonucleotide (IO) used amply an (A) artificial system or (B) the Salmonella InvA gene. The underlined region of the primer is the sequence that is homologous to the IO. The green region of the IO (marked in italics) is modified with 2′-O-methyl RNA. The bold sequence within the IO represents the region that is non-homologous to the target DNA. (PDF) [file pone.0112656.s003.pdf]

# Supporting information S3

# Schematic representation of SIBA primers and the invasion oligonucleotide (IO) used in the (A) artificial and (B) *Salmonella* InvA gene amplification systems.

## A. SIBA artificial system

CTAGAGTCATGTGTAGTTGAC **R-primer**

**TTGTCCATAGACTG**CTCGACCTGATACACGTTATCGTCCATACGGAT*UCGGGAUCUCAUA*/InvdT/**-IO**

AACAAGAAGGCGTACTCGACCTGATACACGTTATCGTCCATACGGATTCGGGATCTCAGTACACATCAACTG-**Target DNA**

AACAAGAAGGCGTACTCGACC **F-primer**

## B. SIBA Salmonella InvA system

GTTGCAAAGGACGCCA-**R-primer**

**TCCTCCTGTACCTTG**TGTTTATGGGGTCGTTCTACATTGACAGAAT*CCUCAGUUUUUCAACGA*/InvdT/**-IO**

TACTGGCGATATTGGTGTTTATGGGGTCGTTCTACATTGACAGAATCCTCAGTTTTTCAACGTTTCCTGCGGTA-**Target DNA**

CTGGCGATATTGGTGTTT **F-primer**

Figure S3. Schematic representation of the SIBA primers and invasion oligonucleotide (IO) used amply an (A) artificial system or (B) the *Salmonella* InvA gene. The underlined region of the primer is the sequence that is homologous to the IO. The highlighted region of the IO (marked in italics) is modified with 2'-O-methyl RNA. The bold sequence within the IO represents the region that is non-homologous to the target DNA (seeding region).
