# Supplementary material for: Strand Invasion Based Amplification (SIBA®): A Novel Isothermal DNA Amplification Technology Demonstrating High Specificity and Sensitivity for a Single Molecule of Target Analyte
Source: PLoS One. 2014 Nov 24;9(11):e112656. doi: 10.1371/journal.pone.0112656 (PMC4242538; doi:10.1371/journal.pone.0112656)
Supplement: Figure S4 — Primer length constraints. (A) Configuration of the forward and reverse primers containing non-homologous regions of different lengths. Blue indicates the region of the target template that is homologous to the IO. The black line on the IO indicates the non-homologous seeding region. The green lines on the IO indicate the 2′-O-methyl RNA region and the black square indicates the inverted dT modification. (B) Real-time monitoring of SIBA reactions with SYBR Green I using various combinations of forward and reverse primers. SB-IO was the IO used in this study. The reactions were performed with 104 copies of SB-long target template. (PDF) [file pone.0112656.s004.pdf]

Supporting information S4

SIBA primer length constraints

Primers used in SIBA are typically designed to be between 16 and 23 nucleotides (nt) in length (depending on the sequence/or melting temperature). Short length ensures that the primers are not substrates for a recombinase (Fig. S1 and S2). The 3'-end of these primers are also designed to be homologous to the IO, such that only about 12–16 nt region of the primers are non-homologous to the IO. The length of this non-homologous region also defines the length of the target duplex region peripheral to the IO invasion site (Fig. 1 and S4).

Here, we showed that the length of the primer non-homologous region (non-homologous to the IO) can impact on the amplification efficiency. If this region is too long the amplification of the target DNA becomes less efficient, or may not occur at all. In this experiment, two forward primers (SB-F21 and SB-F21-nhom-1) and three reverse primers (SB-R21, SB-R21-nhom1, and SB-R21 nhom1) were used in various combinations to amplify a target template (SB-TEMPLATE LONG) in the presence of the IO (SB-IO). These primers differed in the length of their non-homologous regions, and their configurations are described in Figure S4A. Amplification was most efficient when the length of the primer regions that are non-homologous to the IO was 14 nucleotides (SB-F21 and SB-R21). At lengths > 14 nucleotides, amplification was less efficient or completely absent. These observations suggest that there is a limit to the length of the primers that can be used for SIBA, particularly with respect to the regions non-homologous to the IO.

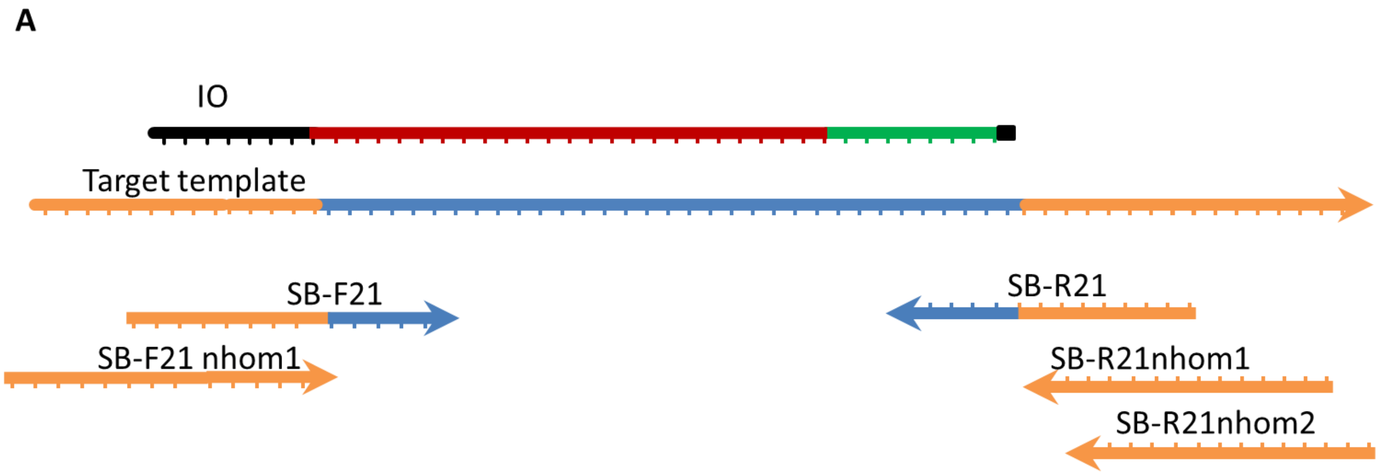

B

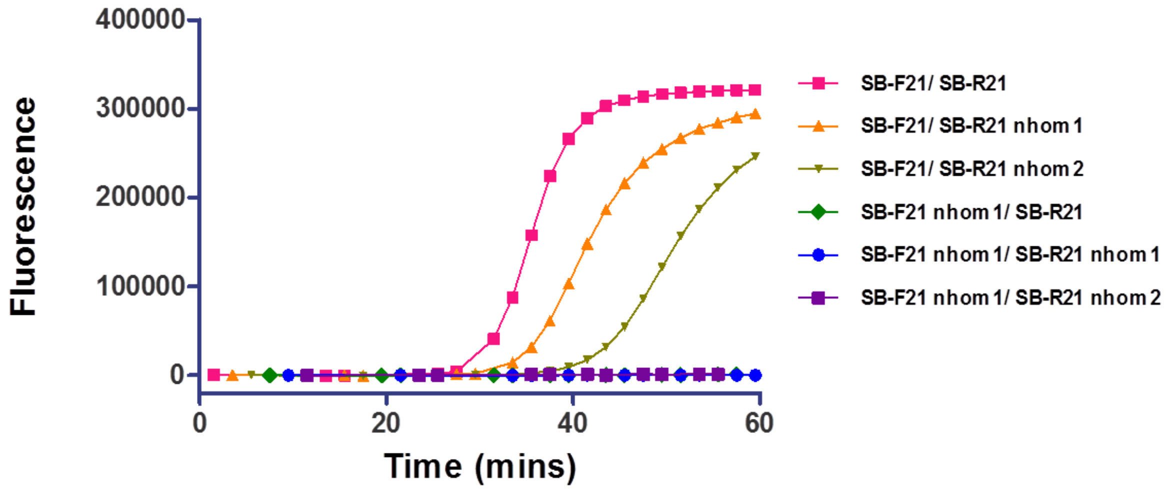

Figure S4. Primer length constraints. (A) Configuration of the forward and reverse primers containing non-homologous regions of different lengths. Blue indicates the region of the target template that is homologous to the IO. The black line on the IO indicates the non-homologous seeding region. The green lines on the IO indicate the 2'-O-methyl RNA region and the black square indicates the inverted dT modification. (B) Real-time monitoring of SIBA reactions with SYBR Green I using various combinations of forward and reverse primers. SB-IO was the IO used in this study. The reactions were performed with 10^4 copies of SB-long target template.
