# Supplementary material for: Strand Invasion Based Amplification (SIBA®): A Novel Isothermal DNA Amplification Technology Demonstrating High Specificity and Sensitivity for a Single Molecule of Target Analyte
Source: PLoS One. 2014 Nov 24;9(11):e112656. doi: 10.1371/journal.pone.0112656 (PMC4242538; doi:10.1371/journal.pone.0112656)
Supplement: Figure S5 — The sensitivity of SIBA is improved by using long primers that are partially homologous to the IO. (A) Configuration of the forward and reverse primers used. The orange line on the primers indicates the region that is non-homologous to the IO. (B) The SIBA reaction was performed using either 104 copies of target DNA (SB-template) or water. Amplification was monitored in real-time using SYBR Green I. The forward primer was either short (non-homologous to the IO; SB-F14) or long (3′-end homologous to the IO; SB-F21). The reverse primer was SB-R21. The IO used was SB-IO. Fig. S5c: Non-specific artifacts are not amplified in the SIBA reaction. Step 1: Primers can non-specifically copy onto the IO; however, this will not include the 2′-O-methyl RNA region since it is not a substrate for polymerase. Step 2: Through a process of strand switching, the product may further copy onto another primer. Step 3: The product formed can have a region homologous to the IO insertion but not to the 2′-O-methyl RNA region. Step 4: The region peripheral to the IO will not dissociate since it is too long to dissociate. (PDF) [file pone.0112656.s005.pdf]

Supporting information 5

Amplification with short primers.

The 3’ and 5’ ends of the primers used in the SIBA are homologous and non-homologous to the IO, respectively. The length of the non-homologous 5’ ends of the primers is typically 14 nucleotides (Fig S3). This configuration ensures efficient amplification of the target DNA and minimizes the risk of non-specific amplification. It is also possible to use short primers ≤ 14 nucleotides in length, which do not have a region homologous to the IO. Here, we demonstrated that primers of this length (≤ 14 nucleotides) can generate non-specific amplification products. A standard SIBA reaction was performed using either a short primer of 14 nucleotides (SB-F14, non-homologous to the IO) or a long primer of 21 nucleotides (SB-F21, with seven nucleotides homologous and 14 nucleotides non-homologous to the IO). SB-R21 and SB-IO were the reverse primer and IO, respectively. Both the short (SB-F14) and long (SB-F21) forward primers were able to amplify the target template efficiently in the presence of the IO. However, the short forward primer (SB-F14) generated non-specific amplification products (Fig. S5B). We proposed that in order for a non-target template to be amplified, it will need to have the IO invasion site and also have a region peripheral to the IO site binding site that is short enough to dissociate. It is plausible that a primer could occasionally extend the DNA region of the IO and subsequently strand switch to copy onto another primer. This could create an amplifiable artifact in which the region peripheral to the IO insertion dissociates during amplification due to the use of short primers. To avoid this problem, we designed longer primers (16–23 nucleotides) with 3’-ends partly homologous to the IO. In this configuration, the region peripheral to the IO is still around 14 nucleotides long (SB-F21). This leaves only a short peripheral region that dissociates when the target DNA is amplified. Conversely, if the longer primer becomes part of an artifactual template, then the peripheral region becomes too long to dissociate (Fig. S5C).

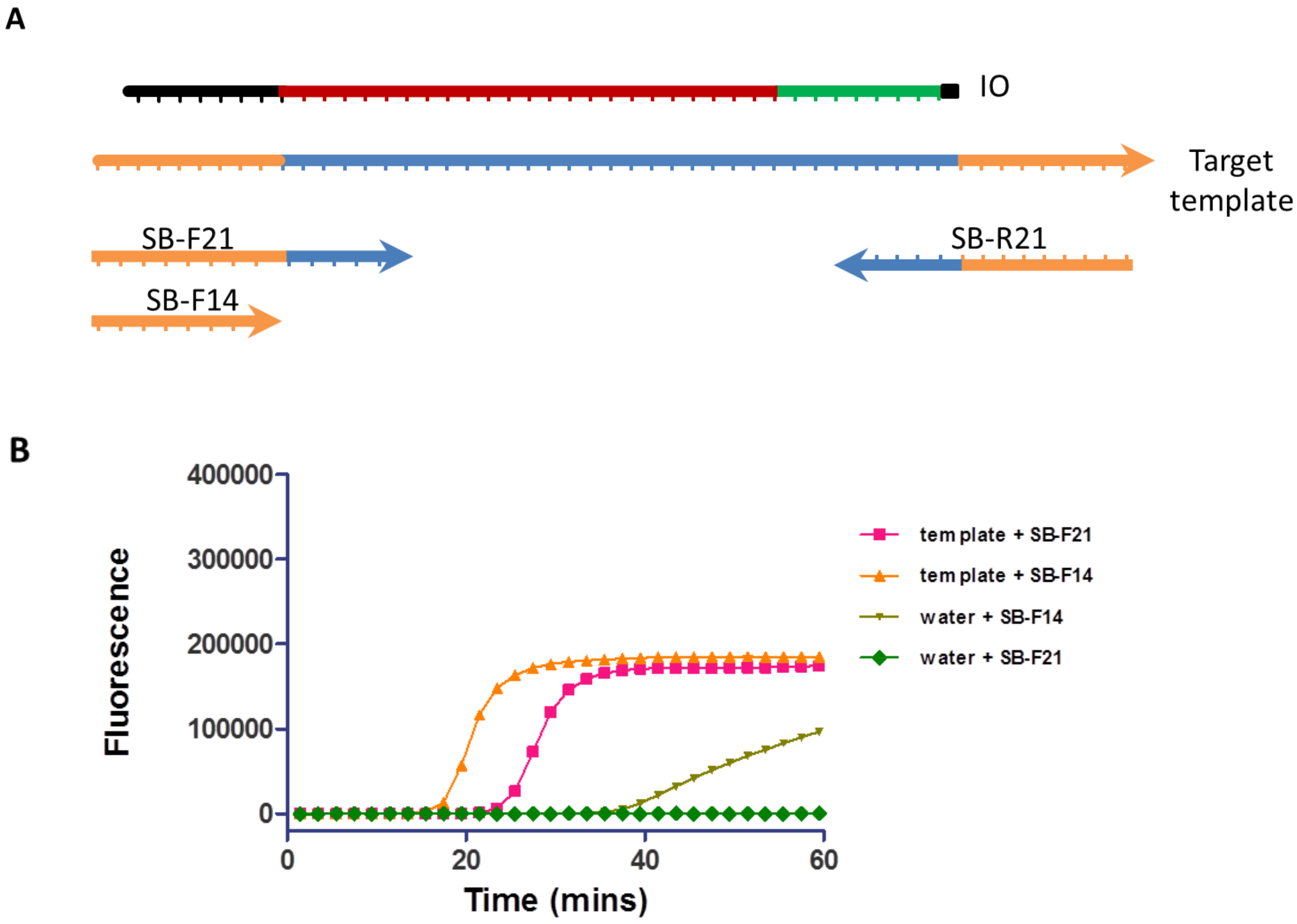

Figure S5. The sensitivity of SIBA is improved by using long primers that are partially homologous to the IO. (A) Configuration of the forward and reverse primers used. The orange line on the primers indicates the region that is non-homologous to the IO. (B) The SIBA reaction was performed using either 10^4 copies of target DNA (SB-template) or water. Amplification was monitored in real-time using SYBR Green I. The forward primer was either short (non-homologous to the IO; SB-F14) or long (3'-end homologous to the IO; SB-F21). The reverse primer was SB-R21. The IO used was SB-IO.

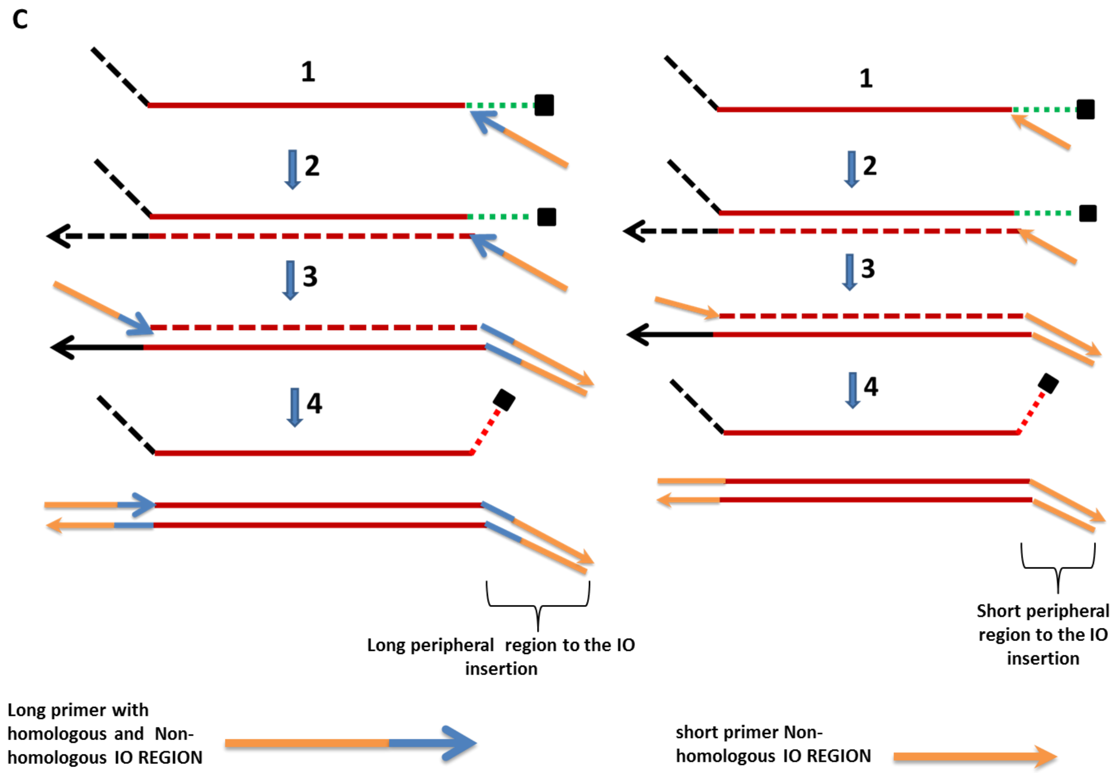

Fig. S5c: Non-specific artifacts are not amplified in the SIBA reaction.

Step 1: Primers can non-specifically copy onto the IO; however, this will not include the 2’-O-methyl RNA region since it is not a substrate for polymerase. Step 2: Through a process of strand switching, the product may further copy onto another primer. Step 3: The product formed can have a region homologous to the IO insertion but not to the 2’-O-methyl RNA region. Step 4: The region peripheral to the IO will not dissociate since it is too long to dissociate.
