# Supplementary material for: Strand Invasion Based Amplification (SIBA®): A Novel Isothermal DNA Amplification Technology Demonstrating High Specificity and Sensitivity for a Single Molecule of Target Analyte
Source: PLoS One. 2014 Nov 24;9(11):e112656. doi: 10.1371/journal.pone.0112656 (PMC4242538; doi:10.1371/journal.pone.0112656)
Supplement: Figure S6 — Sensitivity of the SIBA assay. (A) Amplification of target DNA serially diluted from 106 copies to a single copy (SB-template) per sample. Amplification was monitored in real-time using SYBR Green I. (B) Melting curve analysis of the corresponding reaction products ((-dF (fluorescence)/dT (temperature) versus temperature). SB-F21 and SB-R21 were the forward and reverse primers, respectively. The IO used was SB-IO. (PDF) [file pone.0112656.s006.pdf]

Supporting information 6

SIBA can detect a single copy of target DNA.

The sensitivity of SIBA was investigated using a serially diluted target template (diluted from 10^6 copies to a single copy). The SIBA assay used for detecting an artificial target DNA sequence was used for this purpose (Fig. S3A). The IO, forward and reserve primer used were SB-IO, SB-F21 and SB-R21 respectively. Four independent experiments were performed in triplicate. All reactions that contained ≥ 20 copies of target template were amplified (Fig. S6). The threshold detection time (dt), which was the time at which the SYBR Green I fluorescent signal exceeded the background signal correlated well with the amount of target template used. Two of three samples containing five copies were amplified, while one of three reactions containing two or a single copy was amplified. This is likely due to the presence of target molecules at very low copy number in some samples and their potential binding to the assay wells. All reactions in which amplification was achieved yielded a single amplicon as determined by melting curve analysis. As seen in previous results (Fig. 2), no amplification was observed in the absence of template demonstrating the absence of non-specific amplification.

A

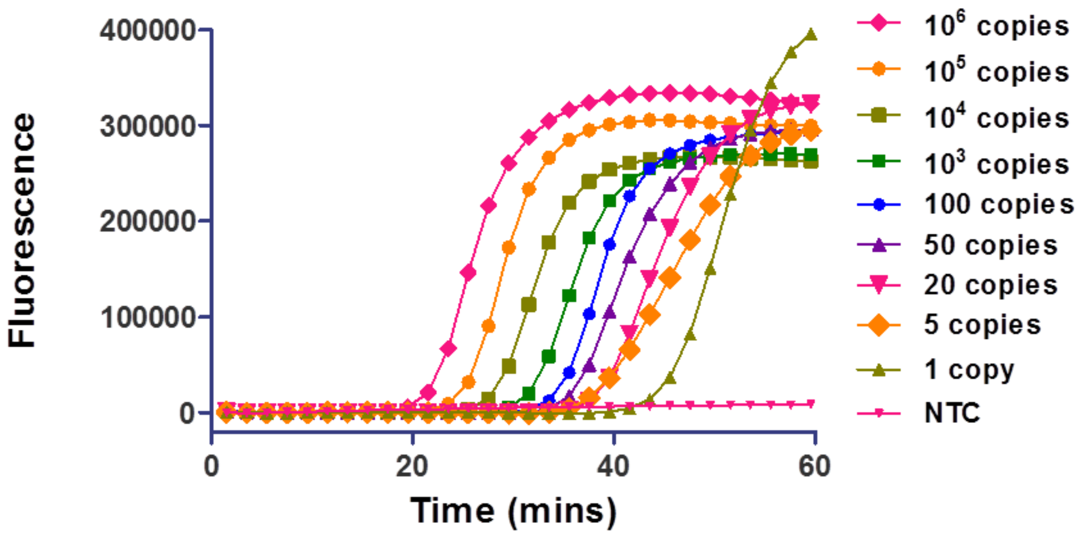

B

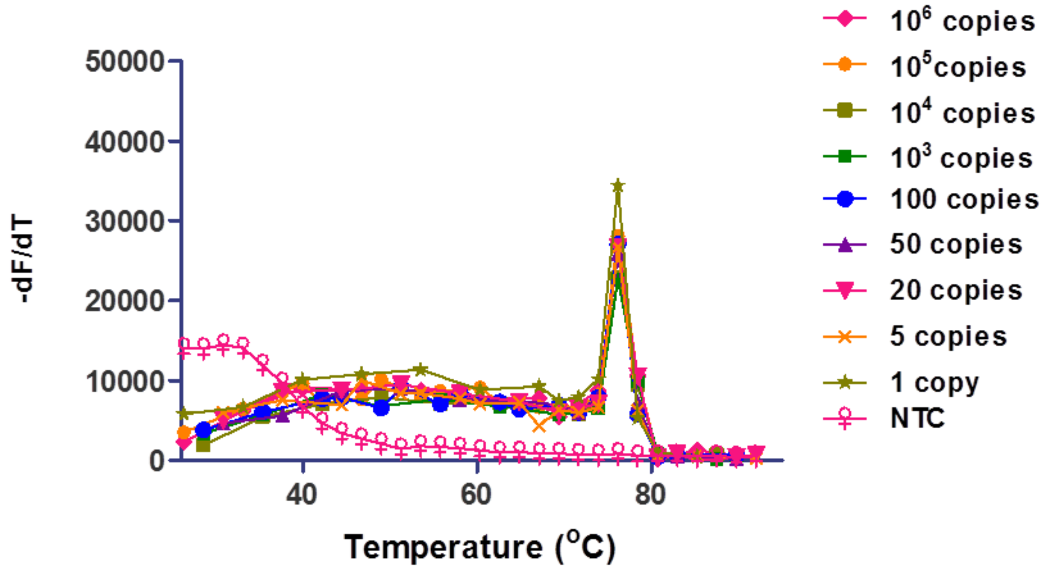

Figure S6. Sensitivity of the SIBA assay. (A) Amplification of target DNA serially diluted from 10^6 copies to a single copy (SB-template) per sample. Amplification was monitored in real-time using SYBR Green I. (B) Melting curve analysis of the corresponding reaction products ((-dF (fluorescence) /dT (temperature) versus temperature). SB-F21 and SB-R21 were the forward and reverse primers, respectively. The IO used was SB-IO.
