# Supplementary material for: Strand Invasion Based Amplification (SIBA®): A Novel Isothermal DNA Amplification Technology Demonstrating High Specificity and Sensitivity for a Single Molecule of Target Analyte
Source: PLoS One. 2014 Nov 24;9(11):e112656. doi: 10.1371/journal.pone.0112656 (PMC4242538; doi:10.1371/journal.pone.0112656)
Supplement: Figure S7 — DNA pairing strand exchange assay of a labeled duplex using different IO configurations. (i) IO fully homologous to the target duplex with a 2′-O-methyl RNA modification, IO-METH. (ii) IO fully homologous to the target duplex with the 2′-O-methyl RNA modification replaced with natural DNA nucleotides, IO-DNA. (iii) IO with a 2′-O-methyl RNA modification that is not homologous to the target duplex, IO DIFF-METH. (iv) IO with the 2′-O-methyl RNA modification deleted, IO-SHORT. Two duplexes, in which the lengths of the downstream region peripheral to the IO differed (14 bp and 25 bp), were used. (PDF) [file pone.0112656.s007.pdf]

Supporting information 7

Partial dissociation of a target duplex.

The 5'-end of primers used in SIBA are non-homologous to the IO and also defines the length of the target duplex region peripheral to IO invasion site. We found that the length of the non-homologous primer region can affect amplification efficiency. When primers with non-homologous region longer than 14 nucleotides were used, amplification was slow or did not occur at all (Fig. S4). We proposed that this could relate to the fact that the target duplex peripheral region should also dissociate upon invasion of target duplex IO binding site. The target duplex region peripheral to the IO would need to be short enough to dissociate in order for complete dissociation of the target duplex to occur. This would then allow target specific primers to bind and extend the target template via the action of a polymerase.

The ability of IO to dissociate homologous labeled DNA duplexes was investigated. Standard SIBA reaction components were used in this experiment, except that the polymerase and dNTPs were excluded. The 3'- and 5'-ends of the duplex were labeled with tetramethylrhodamine (TAMRA) and fluorescein (FAM), respectively, to form a FRET system. Two duplexes, which differed in the lengths of their downstream termini, peripheral to the IO invasion site, were used (the peripheral regions of the short and long duplexes were 14 and 21 bp, respectively). Four different IO configurations were used: (i) IO fully homologous to the target duplex with a 2'-O-methyl RNA modification, IO-METH; (ii) IO fully homologous to the target duplex with the 2'-O-methyl RNA modification replaced with natural DNA nucleotides, IO-DNA; (iii) IO with a 2'-O-methyl RNA modification that was not homologous to the target duplex, IO DIFF-METH; and (iv) IO with a longer 3' end, IO-LONG. The configurations of these IOs and the duplexes used are shown in Figure S7A.

Duplex dissociation was determined by measuring the increase in fluorescein signal generated after the strand labeled with TAMRA was displaced. The experiment was performed by incubating the different IO configurations (100 nM) with 50 nM of labeled short or long duplex. Endpoint fluorescence readings were measured using the Agilent MX pro (Agilent Technologies, Inc., USA) after 20 min at 40°C. The results were represented as a percentage of dissociation by normalizing the FAM signal produced during IO invasion to that generated by a heat denatured (95°C for 10 min) duplex. Here, we showed that complete duplex dissociation is dependent on the length of the

peripheral region of the duplex proximal to the IO. Complete dissociation of the downstream region of the short duplex occurred when the peripheral region corresponded to 14 nucleotides (using IO-DNA, IO-METH, or IO-LONG). The exception was the IO with a non-homologous 2'-O-methyl RNA (IO DIFF-METH). This was because 2'-O-methyl RNA was unable to branch migrate further into the duplex. Such a configuration creates a duplex whose peripheral region becomes longer than 14 nucleotides, thereby limiting its dissociation (Fig. S7B). An increase in the length of the duplex (DUPLEX LONG) results in limited IO-DNA and IO-METH induced dissociation since the peripheral region becomes longer than 14 nucleotides. This suggests that the target duplex region, peripheral to the IO invasion site would need to be approximately 14 base pair so that it is also short enough to dissociate. This would lead to a complete dissociation of the target duplex thus allowing primers to bind efficiently and extend the target via the action of a polymerase. It could explain why primers having non-homologous region longer than 14 nucleotides do not function efficiently in SIBA (Fig S4). Since the length primer non-homologous region determines the target duplex region, peripheral to the IO invasion site.

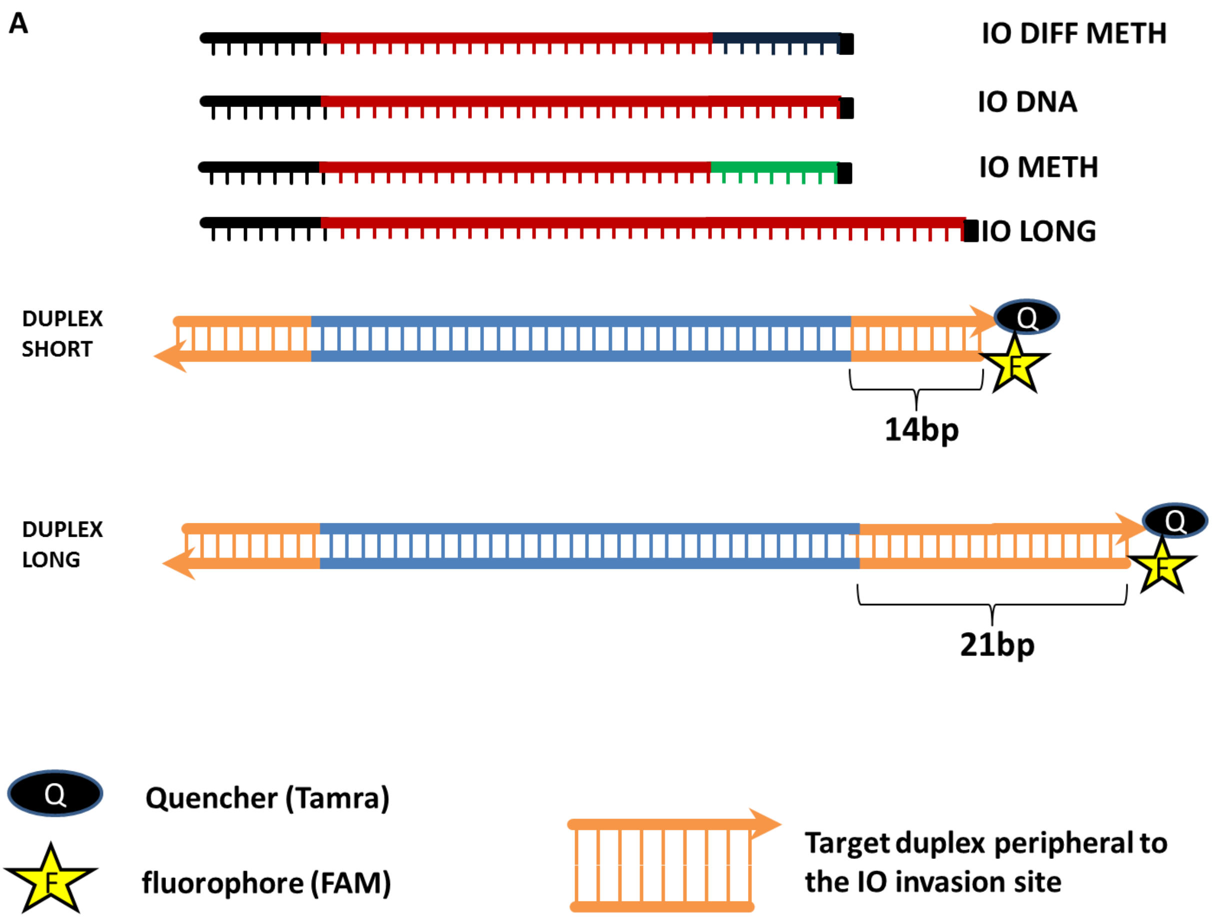

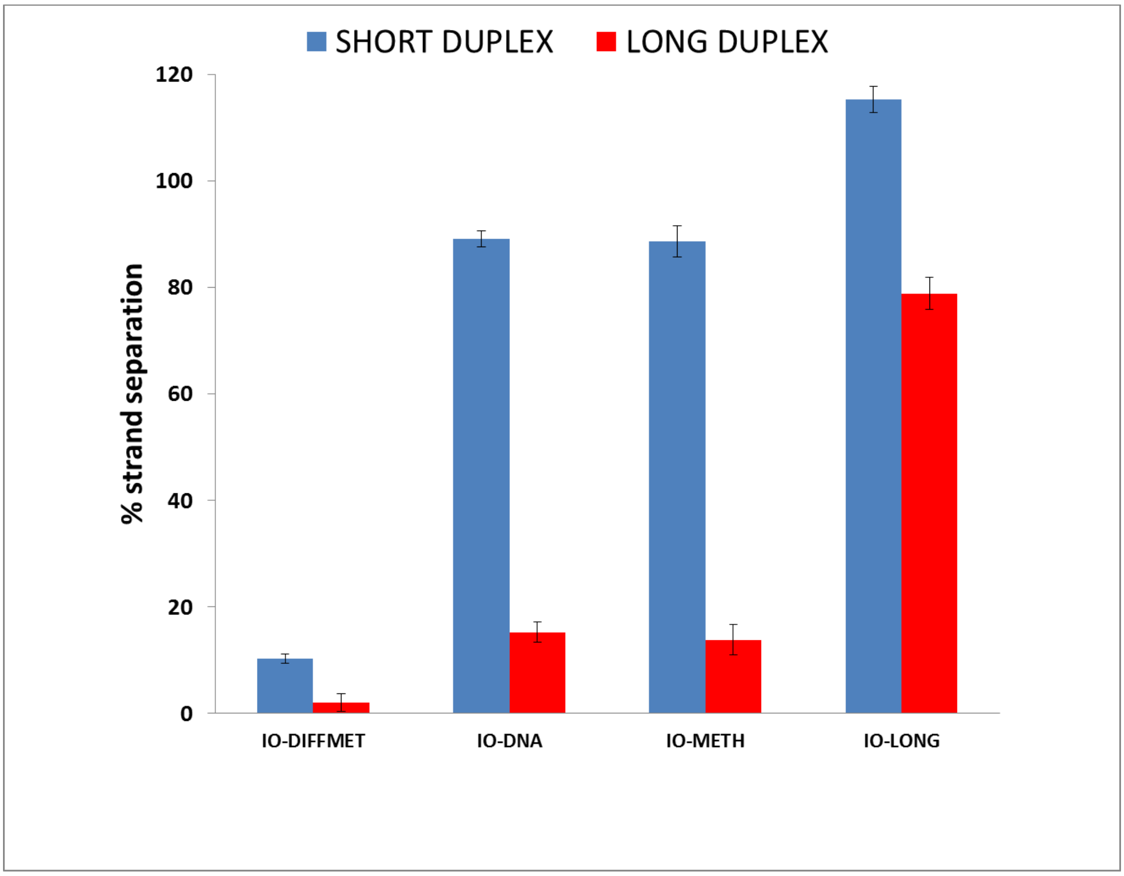

Figure S7. DNA pairing strand exchange assay of a labeled duplex using different IO configurations. (i) IO fully homologous to the target duplex with a 2'-O-methyl RNA modification, IO-METH. (ii) IO fully homologous to the target duplex with the 2'-O-methyl RNA modification replaced with natural DNA nucleotides, IO-DNA. (iii) IO with a 2'-O-methyl RNA modification that is not homologous to the target duplex, IO DIFF-METH. (iv) IO with the 2'-O-methyl RNA modification deleted, IO-SHORT. Two duplexes, in which the lengths of the downstream region peripheral to the IO differed (14 bp and 25 bp), were used.
