# Supplementary material for: Strand Invasion Based Amplification (SIBA®): A Novel Isothermal DNA Amplification Technology Demonstrating High Specificity and Sensitivity for a Single Molecule of Target Analyte
Source: PLoS One. 2014 Nov 24;9(11):e112656. doi: 10.1371/journal.pone.0112656 (PMC4242538; doi:10.1371/journal.pone.0112656)
Supplement: Figure S8 — Minimal lag time associated with the first round of amplification. The SIBA Salmonella assay was performed in the presence or absence of a restriction enzyme, RsaI either with Salmonella genomic DNA or pre-denatured Salmonella genomic DNA (using DMSO). Amplification was monitored using SYBR Green I. (PDF) [file pone.0112656.s008.pdf]

Supporting information 8

Lag time associated with the first round of amplification.

The initial target templates used in Figures 1-4, are artificial target DNA sequences with a predefined IO peripheral region that would be created by the forward and reverse primers. During the first round of amplification, the target DNA duplex completely dissociates upon invasion by the IO, since the region peripheral to the IO binding site is short enough to also dissociate. This allows primers to bind and extend the target template via the action of a polymerase. In contrast, when genomic DNA is used as the initial target template, the duplex cannot be completely dissociated by the IO during the first round of amplification since the region peripheral to the IO invasion site remains double stranded due to its length (Fig. S7). Here, we showed that the complexity of the target genomic DNA did not affect the efficiency of the amplification, but rather a few minutes delay in the threshold detection time (dt) was observed.

Standard SIBA reactions for the *Salmonella* InvA assay were performed according to the protocol described in the “Materials and Methods”. The reactions were performed with either *Salmonella* genomic DNA, or *Salmonella* genomic DNA denatured using 90% DMSO for 5 minutes at room temperature. The denatured genomic DNA was subsequently used in SIBA reactions at dilutions devoid of DMSO. The production of single stranded DNA allows primers to bind and extend the genomic DNA to create a target duplex where either the upstream or downstream region peripheral to the IO site is short enough to dissociate during the first round of amplification. For untreated *Salmonella* genomic DNA, SIBA reaction was performed either in the presence or absence of a restriction enzyme, RsaI (0.1 U/µl) that cleaves sites on the *Salmonella* genomic DNA proximal to the primer binding sites. The inclusion of RsaI to SIBA reaction, creates an initial target duplex, having an upstream and downstream region peripheral to the IO site, short enough to also dissociate during the first round of amplification.

The results are shown in Figure S8. The dt for untreated *Salmonella* genomic DNA performed in the absence of RsaI were a few minutes delayed compared with untreated *Salmonella* genomic DNA performed in the presence of RsaI or DMSO denatured. The dt for 1000 and 100 copies of untreated *Salmonella* genomic DNA performed in the absence of RsaI were 17.5 and 19.5 respectively. While

the dt for 1000 and 100 copies of untreated *Salmonella* genomic DNA performed in the presence of RsaI were 11.5 and 13.5 respectively. The dt for 1000 and 100 copies of DMSO denatured *Salmonella* genomic DNA performed in the absence of RsaI were 15.5 and 17.5 respectively.

However, no significant difference in overall amplification rate was observed amongst untreated, digested or DMSO denatured genomic DNA template as judged by the doubling time (deduced from dt of 1000 vs 100 target copies). This suggests that the slight delay in dt seen with untreated *Salmonella* genomic DNA is probably attributed with the lag time associated with the first round of amplification. Nonetheless, the detection time seen with untreated *Salmonella* genomic DNA is considerable fast even in the absence of RsaI and do not necessitate a pre-denaturation step. SIBA is probably able to overcome the potential problem associated with first round of amplification when genomic DNA is used as the initial target template for the following reasons. Firstly, genomic DNA can exist as negatively supercoiled, and as such can present the target DNA as a single strand template [1]. Secondly, double stranded DNA can occasionally undergo reverse separation and annealing (breathing of the genomic DNA) [1,2,3]. These conditions would allow primers to bind and extend the genomic DNA creating a target duplex that has peripheral region to IO invasion site, short enough to dissociate. In contrast, if primers with long non-homologous IO region are used (Figure S4) or take part in the formation of a non-target template (Figure S5), the duplex created will also also have a peripheral region that will not dissociate during every round of amplification. A delay in first round of amplification and as well as subsequent rounds of amplification takes place. Therefore the doubling time of a non-target template would be much slower than the target template. The non-target template would be unable to amplify efficiently or would not amplify at all.

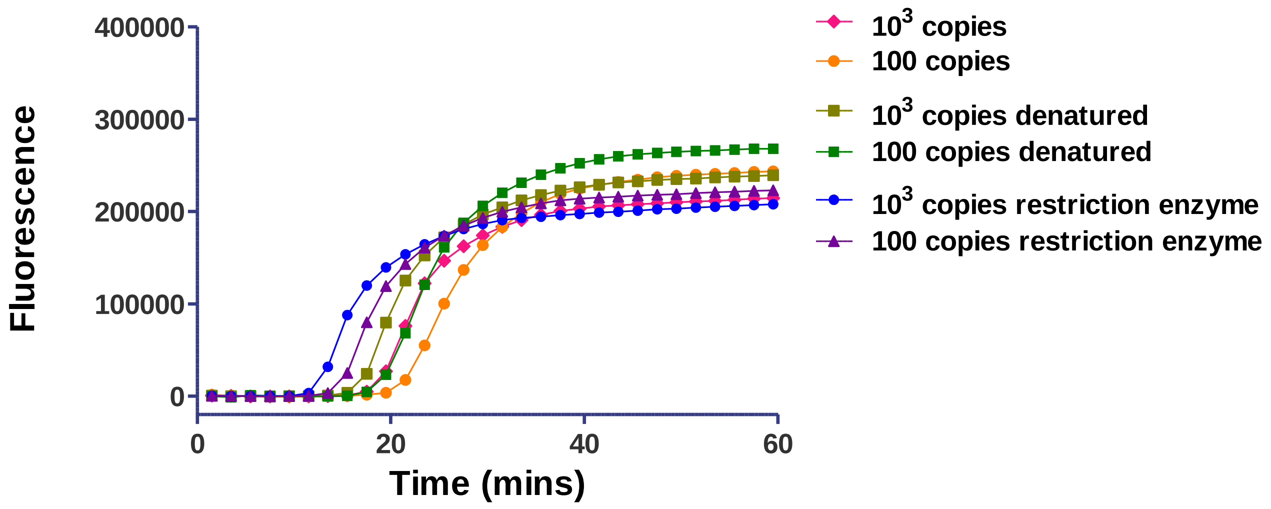

Figure S8. Minimal lag time associated with the first round of amplification. The SIBA *Salmonella* assay was performed in the presence or absence of a restriction enzyme, RsaI either with *Salmonella* genomic DNA or pre-denatured *Salmonella* genomic DNA (using DMSO). Amplification was monitored using SYBR Green I.

References

1. Lilley DMJ (1988) DNA opens up — supercoiling and heavy breathing. Trends in Genetics 4: 111-114.

2. Englander SW, Kallenbach NR, Heeger AJ, Krumhansl JA, Litwin S (1980) Nature of the open state in long polynucleotide double helices: possibility of soliton excitations. Proceedings of the National Academy of Sciences 77: 7222-7226.

3. Gueron M, Kochoyan M, Leroy JL (1987) A single mode of DNA base-pair opening drives imino proton exchange. Nature 328: 89-92.
