# Supplementary material for: Strand Invasion Based Amplification (SIBA®): A Novel Isothermal DNA Amplification Technology Demonstrating High Specificity and Sensitivity for a Single Molecule of Target Analyte
Source: PLoS One. 2014 Nov 24;9(11):e112656. doi: 10.1371/journal.pone.0112656 (PMC4242538; doi:10.1371/journal.pone.0112656)
Supplement: Table S1 — Oligonucleotides used in this study. (PDF) [file pone.0112656.s009.pdf]

Table S1. Oligonucleotides used in this study

Primers

Primers for the SIBA artificial system

SB-F30	TGCCAATCCAACAAGAAGGCGTACTCGACC
SB-F35	CAATTTGCCAATCCAACAAGAAGGCGTACTCGACC
SB-F21	AACAAGAAGGCGTACTCGACC
SB-F21 NHOM1	TGCCAATCCAACAAGAAGGCG
SB-F18	CAAGAAGGCGTACTCGAC
SB-F16	CAAGAAGGCGTACTCG
SB-F17	TGATGTGTACTGAGATC
SB-F14	AACAAGAAGGCGTA
SB-R30	GCAATGAGCTCAGTTGATGTGTACTGAGATC
SB-R35	ACCATGCAATGAGCTCAGTTGATGTGTACTGAGATC
SB-R21	CAGTTGATGTGTACTGAGATC
SB-R21 NHOM1	ATGAGCTCAGTTGATGTGTAC
SB-R21 NHOM2	GCAATGAGCTCAGTTGATGTG
SB-R18	TTGATGTGTACTGAGATC
SB-R16	TTGATGTGTACTGAGA
SB-R14	TGTGTACTGAGATC

Primers used for the *Salmonella* system

SM-F18 CTGGCGATATTGGTGTTT

SM-R16 ACCGCAGGAAACGTTG

Regions homologous to the IO are underlined.

Templates

Template used for the SIBA artificial system

SB-TEMPLATE LONG

CAATTTGCCAATCCAACAAGAAGGCGTACTCGACCTGATACACGTTATCGTCCATACGGATTCGGG
ATCTCAGTACACATCAACTGAGCTCATTGCATGGT

SB-TEMPLATE

AACAAGAAGGCGTACTCGACCTGATACACGTTATCGTCCATACGGATTCGGGATCTCAGTACACAT
CAACTG

Non-homologous template used for the SIBA artificial system

SB NHOM Template

TACTGGCGATATTGGTGTTTATGGCGTCCTTCTACAAAGACAGAATCCTCTCTTTTTCAACGTTTC
CTGCGGTA

Templates (harboring point mutations) used for the SIBA artificial system specificity test

Point mutations located in regions homologous to the IO 2'-O-methyl RNA region

SB-MET1 TEMPLATE

AACAAGAAGGCGTACTCGACCTGATACACGTTATCGTCCATACGGATTC**C**GGATCTCAGTACACATCAACTG

SB-MET2 TEMPLATE

AACAAGAAGGCGTACTCGACCTGATACACGTTATCGTCCATACGGATTC**CC**GATCTCAGTACACATCAACTG

SB-MET3 TEMPLATE

AACAAGAAGGCGTACTCGACCTGATACACGTTATCGTCCATACGGATT**GCC**GATCTCAGTACACATCAACTG

SB-MET4 TEMPLATE

AACAAGAAGGCGTACTCGACCTGATACACGTTATCGTCCATACGGAT**TGCC**GATCTCAGTACACATCAACTG

Point mutations located in regions homologous to the IO-DNA region

SB-DNA1 TEMPLATE

AACAAGAAGGCGTACTCGACCT**C**ATACACGTTATCGTCCATACGGATTCGGGATCTCAGTACACATCAACTG

SB-DNA2 TEMPLATE

AACAAGAAGGCGTACTCGACCT**C**ATACACGTTATCGTCC**T**TACGGATTCGGGATCTCAGTACACATCAACTG

SB-DNA3 TEMPLATE

AACAAGAAGGCGTACTCGACCT**C**ATACAC**C**TTATCGTCC**T**TACGGATTCGGGATCTCAGTACACATCAACTG

SB-DNA4 TEMPLATE

AACAAGAAGGCGTACTCGACCT**C**ATACAC**C**TTA**A**CGTCC**T**TACGGATTCGGGATCTCAGTACACATCAACTG

Point mutations are underlined and in bold.

Invasion oligonucleotide (IO) sequences used in the SIBA artificial system

IO used for the artificial system

SB-IO

TTGTCCATAGACTG CTCGACCTGATACACGTTATCGTCCATACGGAT mUmCmGmGmGmAmUmCmUmCmAmUmA/InvdT/

SB-IO NON-METH

TTGTCCATAGACTG CTCGACCTGATACACGTTATCGTCCATACGGAT TCGG TA /InvdT/

SB-IO-DNA

TTGTCCATAGACTG CTCGACCTGATACACGTTATCGTCCATACGGATTCGGGATCTCA TA /InvdT/

SB-IO DIFF-METH

TTGTCCATAGACTG CTCGACCTGATACACGTTATCGTCCATACGGAT mAmGmCmCmCmUmCmAmGmAmGmUmA /InvdT/

The IO non-homologous region (5'-end) is underlined.

m = 2'-O-methyl RNA nucleotides.

/InvdT/ = inverted dT.

Non-homologous IO used for specificity evaluation

SB NHOM IO

TCCTCCTGTACCTT GTGTTTATGGGGTCGTTCTACATTGACAGAATCCT mCmAmGmUmUmUmUmUmCmAmAmCmGmA/InvdT/

IO non-homologous region (5'-end) is underlined.

m = 2'-O-methyl RNA nucleotides.

/InvdT/ = inverted dT.

Invasion oligonucleotide (IO) sequence used for the *Salmonella* system
**SM-IO**
TCCTCCTCTTCCTT TGTTTATGGGGTCGTTCTACATTGACAGAATC mCmUmCmAmGmUmUmUmUmUmCmAmAmCmGmA/InvdT/<br><br>The IO non-homologous region (5'-end) is underlined.<br>m = 2'-O-methyl RNA nucleotides.<br>/InvdT/ = inverted dT.

Oligonucleotides used in the strand exchange experiment	
SX 16	*GAGTACACATGAAGTG*
SX 21	*CTACTGAGTACACATGAAGTG*
SX 25	*AGCCCTACTGAGTACACATGAAGTG*
SX 35	*GCATACCGATAGCCCTACTGAGTACACATGAAGTG*
SX 40	*ATCGTGCATACCGATAGCCCTACTGAGTACACATGAAGTG*
SX 49	*ATAGACGTAATCGTGCATACCGATAGCCCTACTGAGTACACATGAAGTG*

SX 16–49 were labeled (or not) with TAMRA at the 3'-end.

INVASION TEMPLATE

/FAM/CACTTCATGTGTACTCAGTAGGGCTATCGGTATGCACGATTACGTCTATG

Invasion oligonucleotide (IO) sequence used for the invasion strand exchange assay

IO-LONG

TTGTCCATAGACTG CTGCACCTCATAGACGTAATCGTGCATACCGATAGCCCTACTGA GTACACATGA TA /InvdT/

IO-DNA

TTGTCCATAGACTG CTGCACCTCATAGACGTAATCGTGCATACCGATAGCCCTACTGATA

/InvdT/

IO-MET

TTGTCCATAGACTG CTGCACCTCATAGACGTAATCGTGCATACCGAT

AmGmCmCmCmTmAmCmTmGmAmTmA /InvdT/

IO DIFF-MET

TTGTCCATAGACTG CTGCACCTCATAGACGTAATCGTGCATACCGAT A

mCmAmUmGmAmUmGmAmAmGmCmU /InvdT/

The IO non-homologous region (5'-end) is underlined.

m = 2'-O-methyl RNA nucleotides.

/InvdT/ = inverted dT.

Duplex used for the invasion strand exchange assay

Duplex SHORT

AACAAGAAGGCGTACTGCACCTCATAGACGTAATCGTGCATACCGATAGCCCTACTGAGTACACAT

GAAGTG/TAMRA/

/FAM/CACTTCATGTGTACTCAGTAGGGCTATCGGTATGCACGATTACGTCTATGAGGTGCAGTAC

GCCTTCTTGTT

Duplex LONG

AACAAGAAGGCGTACTGCACCTCATAGACGTAATCGTGCATACCGATAGCCCTACTGAGTACACAT

GAAGTGAGTTCAGCAG/TAMRA/

/FAM/CTGCTGAACTCACTTCATGTGTACTCAGTAGGGCTATCGGTATGCACGATTACGTCTATGA

GGTGCAGTACGCCTTCTTGTT
