# Supplementary material for: Strand Invasion Based Amplification (SIBA®): A Novel Isothermal DNA Amplification Technology Demonstrating High Specificity and Sensitivity for a Single Molecule of Target Analyte
Source: PLoS One. 2014 Nov 24;9(11):e112656. doi: 10.1371/journal.pone.0112656 (PMC4242538; doi:10.1371/journal.pone.0112656)
Supplement: Table S2 — Bacterial strains used in this study. (PDF) [file pone.0112656.s010.pdf]

Table S2. Bacterial strains used in this study

***Salmonella* strain used for the SIBA *Salmonella* assay sensitivity test**	**Bacterial mix used for the SIBA *Salmonella* assay specificity test**
Salmonella typhimurium ATCC 14028	*Enterobacter aerogenes* ATCC13048<br>*Citrobacter sp.*<br>*Shigella sonnei* ATCC25931<br>*Shigella flexneri*<br>*Streptococcus agalactiae* (B) ATCC12386<br><br>*Streptococcus agalactiae* (B) ATCC27956<br>*Listeria monocytogenes* NCTC11994<br>*Escherichia coli* ATCC25922<br>*Enterobacter aerogenes* ATCC15038<br>*Enterobacter cloacae* 118/1986<br><br>*Enterobacter aerogenes* NCTC1006<br>*Enterobacter spp.* (Paper Mill isolate)<br>*Enterococcus faecalis* ATCC29212<br>*Citrobacter freundii* ATCC8090<br>*Klebsiella pneumoniae* ATCC13883
